# CD9 regulates macrophage-mediated remodeling of adipose tissue in obesity

**DOI:** 10.1172/jci.insight.193837

**Published:** 2026-02-10

**Authors:** Julia Chini, Nicole DeMarco, Dana V. Mitchell, Sam J. McCright, Kaitlyn M. Shen, Divyansi Pandey, Rachel L. Clement, Jessica Miller, Rajan Jain, Deanne M. Taylor, Mitchell A. Lazar, David A. Hill

**Affiliations:** 1 Department of Pediatrics, Perelman School of Medicine, University of Pennsylvania, Philadelphia, Pennsylvania, USA.; 2 Division of Allergy and Immunology, Children’s Hospital of Philadelphia, Philadelphia, Pennsylvania, USA.; 3 Institute for Immunology and Immune Health, Perelman School of Medicine, University of Pennsylvania, Philadelphia, Pennsylvania, USA.; 4 Department of Biomedical and Health Informatics, Children’s Hospital of Philadelphia, Philadelphia, Pennsylvania, USA.; 5 Departments of Medicine and Cell and Developmental Biology, Penn Cardiovascular Institute, Penn Epigenetics Institute,; 6 Department of Medicine, Division of Endocrinology, and; 7 Institute for Diabetes, Obesity and Metabolism, Perelman School of Medicine, University of Pennsylvania, Philadelphia, Pennsylvania, USA.

**Keywords:** Immunology, Metabolism, Adipose tissue, Macrophages

## Abstract

Dysfunctional white adipose tissue contributes to the development of obesity-related morbidities, including insulin resistance, dyslipidemia, and other metabolic disorders. Adipose tissue macrophages (ATMs) accumulate in obesity and play both beneficial and harmful roles in the maintenance of adipose tissue homeostasis and function. Despite their importance, the molecules and mechanisms that regulate these diverse functions are not well understood. Lipid-associated macrophages (LAMs), the dominant subset of obesity-associated ATMs, accumulate in crown-like structures and are characterized by a metabolically activated and proinflammatory phenotype. We previously identified CD9 as a surface marker of LAMs. However, the contribution of CD9 to the activation and function of LAMs during obesity is unknown. Using a myeloid-specific CD9-KO model, we show that CD9 supports ATM-adipocyte adhesion and crown-like structure formation. Furthermore, CD9 promotes the expression of profibrotic and extracellular matrix remodeling genes. Loss of myeloid CD9 reduces adipose tissue fibrosis, increases visceral adipose tissue accumulation, and improves global metabolic outcomes during diet-induced obesity. These results identify CD9 as a causal regulator of pathogenic LAM functions, highlighting CD9 as a potential therapeutic target for treating obesity-associated metabolic disease.

## Abstract 2

CD9 regulates adipose tissue macrophage adhesion and pro-fibrotic activation. Deleting CD9 improves adipose tissue fibrosis and global metabolism during obesity, identifying it as a potential therapeutic target.

## Graphical abstract

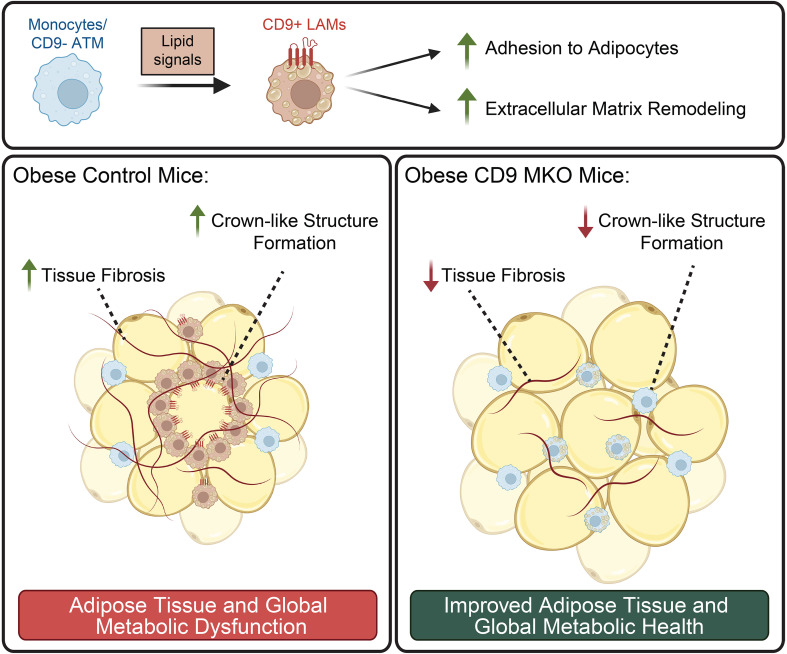

## Introduction

White adipose tissue undergoes dramatic remodeling in response to excess caloric intake, resulting in tissue expansion, inflammation, and fibrosis. This pathological remodeling promotes insulin resistance and ectopic lipid deposition in peripheral organs, predisposing individuals to the development of metabolic sequelae such as metabolic dysfunction–associated fatty liver disease, atherosclerosis, and type 2 diabetes (1). Although newer therapeutics, including glucagon-like peptide-1 (GLP-1) receptor agonists, can induce meaningful weight loss, emerging evidence indicates that the inflammatory and fibrotic features of adipose tissue remodeling often persist (2–6). Defining the mechanisms that drive adipose tissue remodeling is therefore essential for developing more effective strategies to prevent and treat metabolic disease.

In response to excess caloric intake, adipose tissue initially undergoes adaptive expansion through adipocyte hypertrophy and hyperplasia, thereby increasing its storage capacity for lipids. However, with prolonged overnutrition, adipocyte hypertrophy can outpace angiogenesis, leading to tissue hypoxia and adipocyte death (7). Adipocyte dysfunction and apoptosis promote the infiltration and proliferation of immune cells, particularly adipose tissue macrophages (ATMs). ATMs accumulate in crown-like structures surrounding dead and dying adipocytes, where they support tissue homeostasis through the efferocytosis of apoptotic adipocytes and the uptake of free fatty acids released during adipocyte lipolysis (8–12). At the same time, ATMs can contribute to adipose tissue dysfunction by producing pro-inflammatory and profibrotic mediators (10, 13–19). Despite their central role in adipose tissue remodeling, the molecular pathways that direct beneficial versus pathogenic ATM functions remain poorly understood.

During obesity, a subset of ATMs acquires a metabolically activated phenotype characterized by increased lipid accumulation and processing, fatty acid–dependent cellular metabolism, and proinflammatory cytokine production (9, 10, 13, 14, 20). This population, termed lipid-associated macrophages (LAMs), arises largely from recruited monocytes and becomes the dominant ATM subset in adipose tissue during obesity (19). LAMs localize to crown-like structures surrounding dead and dying adipocytes, where they mediate efferocytosis and lipid handling in part through the lipid receptor TREM2 (11, 21, 22).

We previously identified CD9 as a surface marker of LAMs in both mice and humans (11, 23). CD9 is a tetraspanin molecule that recruits and stabilizes proteins such as integrins, cytokine receptors, and metalloproteinases to tetraspanin-enriched microdomains (24–28). Through these interactions, CD9 contributes to cellular adhesion, migration, signal transduction, and extracellular vesicle formation (24, 26, 29–32). Since our initial report, CD9 has been widely used to identify LAMs across multiple other tissues, including the liver and aorta (33–38). Scar-associated macrophages (SAMs), which accumulate during liver and lung fibrosis, also share features with LAMs, including the expression of CD9 (36, 37, 39, 40). Despite the strong association between macrophage CD9 expression and metabolic dysfunction, the influence of CD9 on LAM functions during obesity remains unknown.

To investigate the functional role of CD9 in LAMs during obesity, we developed and characterized a myeloid-specific CD9-KO mouse model. Using this model, we identified a role for CD9 in mediating macrophage adhesion to adipocytes, which is critical for crown-like structure formation during obesity. Moreover, we found that CD9 promotes the expression of fibrosis-associated genes in macrophages. Consequently, loss of CD9 in myeloid cells led to attenuated adipose tissue remodeling and fibrosis, resulting in improved systemic metabolic outcomes during diet-induced obesity. Together, these findings establish CD9 as a pathogenic regulator of LAM function, suggesting that CD9 may be a potential therapeutic target for obesity-associated metabolic disease.

## Results

### CD9^+^ ATMs display a transcriptional profile enriched for extracellular matrix remodeling and adhesion pathways.

**Figure 1:**
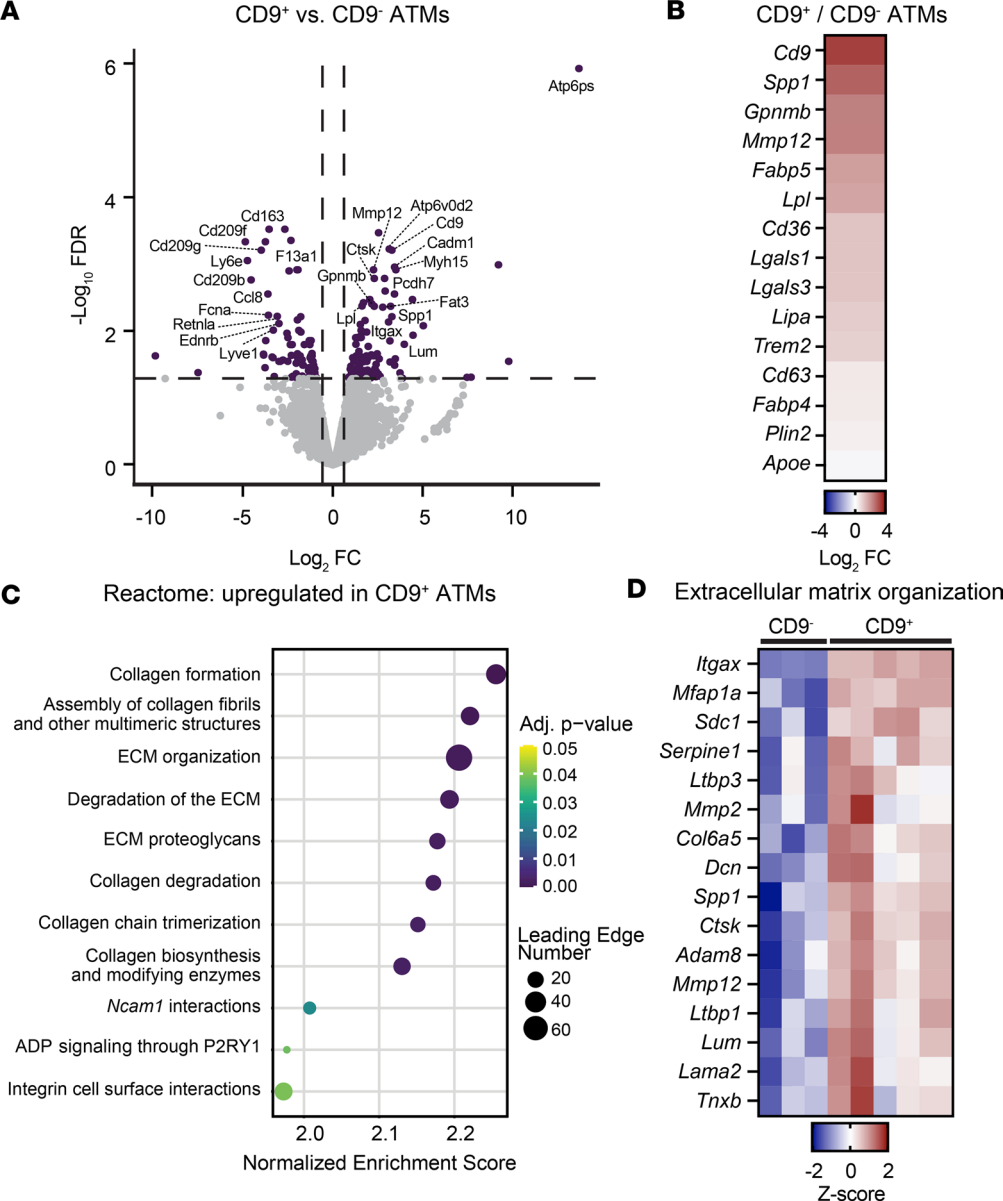
CD9^+^ adipose tissue macrophages have increased expression of extracellular matrix remodeling and adhesion genes. Bulk RNA-Seq analysis of CD9^+^ and CD9^–^ adipose tissue macrophages (ATMs) sorted from epididymal white adipose tissue (eWAT) of WT male mice fed an HFD for 12 weeks (*n* = 3–5). (**A**) Volcano plot of gene expression in CD9^+^ versus CD9^–^ ATMs showing the log_2_ fold-change (FC; *x* axis) and adjusted *P* value (–log_10_ FDR; *y* axis) of genes. Significantly differentially expressed genes (log_2_ FC > 0.58 and FDR < 0.05) are shown in purple. (**B**) Differential expression of genes previously associated with CD9^+^ macrophages (log_2_ FC of CD9^+^ ATMs/CD9^–^ ATMs). Genes are listed in order from highest to lowest log_2_ FC. (**C**) Gene set enrichment analysis showing all Reactome pathways significantly upregulated in CD9^+^ ATMs compared with CD9^–^ ATMs. (**D**) Heatmap of differentially expressed genes (FDR > 0.1) from Extracellular Matrix Organization Pathway (Reactome: R-MMU-1474244). P2RY1, p2Y purinoreceptor 1.

We previously compared the transcriptional profiles of CD9^+^ ATMs and Ly6c^+^ monocytes and found that CD9^+^ ATMs were enriched for proinflammatory and lysosomal-dependent lipid metabolism gene programs (23). During obesity, CD9^+^ ATMs are thought to arise from monocytes via a CD9^–^ ATM transitional state (11, 41). To further investigate the role of CD9 in ATMs, we compared the transcriptomes of sort-purified CD9^+^ and CD9^–^ ATMs isolated from the visceral epididymal white adipose tissue (eWAT) of WT mice fed a high-fat diet (HFD) for 12 weeks (23). We found that 97 genes were upregulated and 84 genes were downregulated in CD9^+^ ATMs compared with CD9^–^ ATMs (Figure 1A). CD9^–^ ATMs demonstrated increased expression of genes encoding pattern recognition receptors (*Cd209b*, *Cd209f*, and *Cd209g)* and genes associated with antiinflammatory and perivascular macrophages (*Lyve1*, *Retnla*, *Cd163*, and *Ednrb*) (42). In contrast, CD9^+^ ATMs were enriched for genes previously identified in LAMs and SAMs, including *Spp1*, *Gpnmb*, *Mmp12*, *Fabp5*, *Lpl*, *Cd36*, and *Lgals3* (9, 11, 13, 14, 36, 43) (Figure 1, A and B).

Gene set enrichment analysis revealed that CD9^+^ ATMs were enriched for pathways involved in extracellular matrix (ECM) remodeling, including programs related to both ECM formation and degradation (Figure 1, C and D). Furthermore, CD9^+^ ATMs were enriched for genes associated with cell adhesion, including *Ncam1* (CD56) and integrin-related interactions (Figure 1C). These data indicate that CD9^+^ ATMs have a transcriptional profile characterized by enhanced expression of ECM-remodeling and adhesion-associated genes.

### CD9 stabilizes ATM-adipocyte interactions in vivo.

During obesity, CD9^+^ LAMs accumulate in adipose tissue in crown-like structures surrounding dead or dying adipocytes (44–46). We therefore investigated whether CD9 regulates ATM accumulation and localization in adipose tissue during obesity. To assess the functional role of CD9 in macrophages, we first generated a *Cd9* conditional KO mouse line (*Cd9^fl/fl^*) by inserting *loxP* sites in the endogenous *Cd9* locus (Supplemental Figure 1A; supplemental material available online with this article; https://doi.org/10.1172/jci.insight.193837DS1). *Cd9^fl/fl^* mice were then crossed to mice expressing Cre recombinase under the endogenous *Lyz2* promoter (*LysMCre*) to generate a myeloid-specific CD9-KO (CD9-MKO) mouse model (47). Successful insertion of *loxP* sites was confirmed by DNA PCR genotyping (Supplemental Figure 1B). A reduction in gene expression of *Cd9* and cell-surface expression of CD9 was confirmed by qPCR and flow cytometry of unpolarized BM-derived macrophages (BMDMs) (Supplemental Figure 1, C and D). Because CD9-MKO BMDMs exhibited only partial CD9 deletion, we also crossed *Cd9^fl/fl^* mice with Vav-iCre mice to generate a hematopoietic-wide CD9 KO (48). BMDMs from *Cd9^fl/fl^* *Vav-iCre*^+/–^ mice had complete ablation of CD9 expression, thereby confirming the efficiency and fidelity of the *Cd9^fl/fl^* conditional allele (Supplemental Figure 1, E and F).

**Figure 2:**
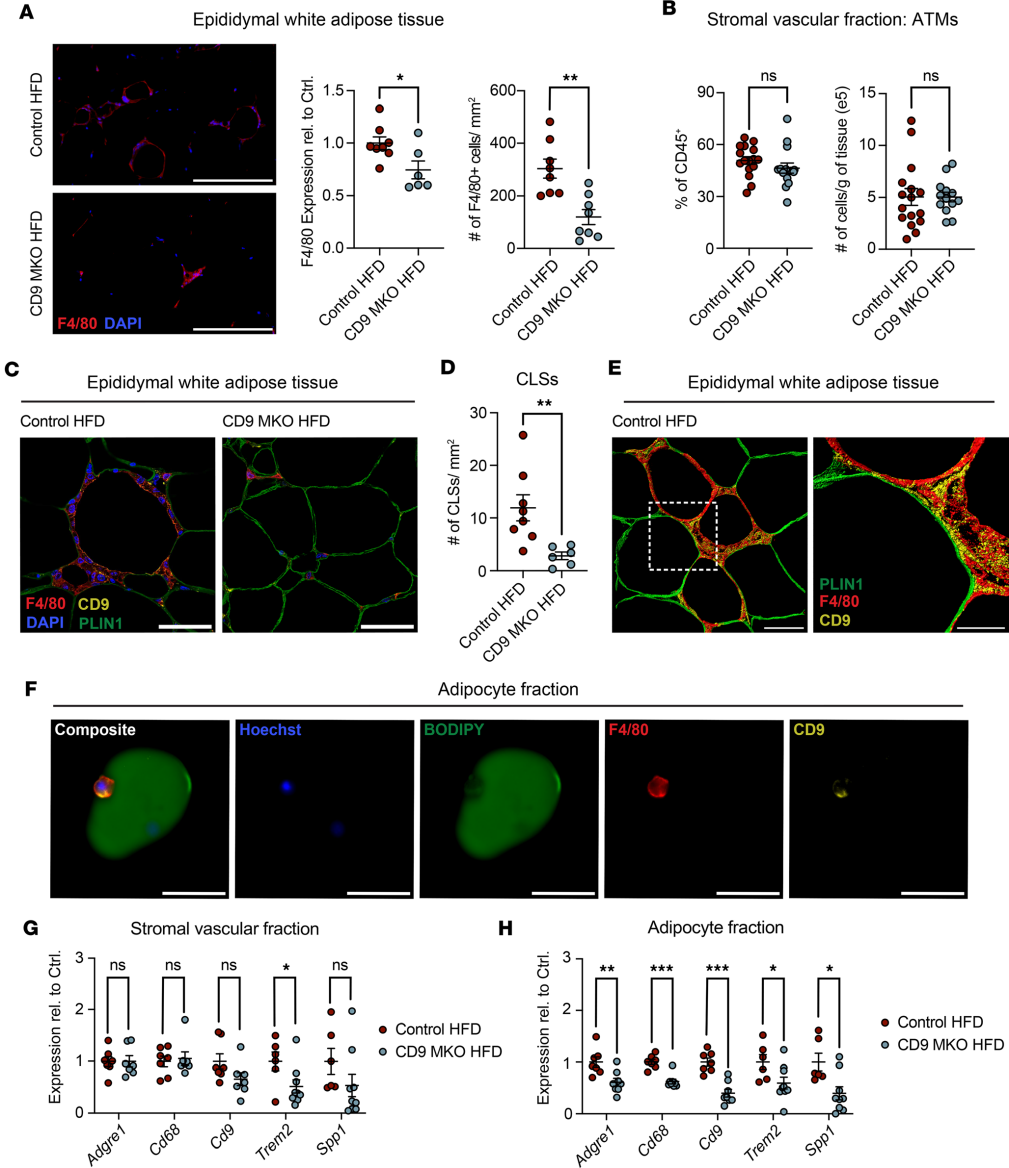
CD9 promotes adipose tissue macrophage accumulation, crown-like structure formation, and adipocyte interactions. Analysis of epididymal white adipose tissue (eWAT) from control (*Cd9^fl/fl^*) and CD9-MKO (*Cd9^fl/fl^ LysMCre*^+/–^) male mice fed an HFD for 12 weeks. (**A**) Representative images and quantification of immunofluorescence staining of F4/80 and DAPI in eWAT tissue sections. Data shown as relative MFI of F4/80 (*n* = 6–8) and number of F4/80^+^ DAPI^+^ cells per mm^2^ (*n* = 8). Scale bar: 200 μm. (**B**) Quantification by flow cytometry of adipose tissue macrophages (ATMs) from control and CD9-MKO HFD mice. Data are shown as frequency of total CD45^+^ immune cells (*n* = 15–16) and cells per gram of tissue (*n* = 13–16). (**C**) Representative high-resolution images of eWAT tissue sections from control and CD9-MKO HFD mice stained with DAPI (blue), PLIN1 (green), F4/80 (red), and CD9 (yellow) (*n* = 8). Scale bar: 50 μm. **(D**) F4/80^+^ crown-like structures (CLSs) per mm^2^ quantified from images from samples shown in **A** (*n* = 6–8). (**E**) 3D image reconstruction of PLIN1 (green), F4/80 (red), and CD9 (yellow) from control HFD mice (*n* = 8). Scale bar: 50 μm (left) or 20 μm (right). (**F**) Representative images of adipocyte fraction isolated from control HFD mice eWAT and stained for Hoechst (blue), BODIPY (green), F4/80 (red), and CD9 (yellow) (*n* = 3). Scale bar: 50 μm. (**G** and **H**) Relative expression of *Adgre1* (F4/80), *Cd68*, *Cd9*, *Trem2*, and *Spp1* (OPN) in stromal vascular fraction (*n* = 6–9; **G**) and adipocyte fraction (*n* = 6–9; **H**) isolated from eWAT. Data are from 2 (**A**, **B**, **D**, and **E**) or 3 (**C**, and **F**–**H**) independent pooled experiments. Data presented as mean ± SEM (**A**–**C**, **G**, and **H**). qPCR shown as ΔCt relative to a normalization factor relative to control HFD samples. Unpaired 2-tailed Student’s *t* test (**A**–**C**, **G**, and **H**). ns, not significant; *P < 0.05, **P < 0.01, ***P < 0.001.

Next, we placed CD9-MKO and control (*Cd9^fl/fl^*) mice on an HFD for 12 weeks and examined the visceral eWAT depot. Since female mice are known to be resistant to adipose tissue inflammation during diet-induced obesity (49–51), initial studies were performed in male mice. Immunofluorescence staining of eWAT sections revealed decreased expression of the pan-macrophage markers F4/80 and CD68 in CD9-MKO HFD mice compared with control HFD mice. In addition, the number of F4/80^+^ and CD68^+^ cells were also reduced in CD9-MKO HFD mice compared with control HFD mice (Figure 2A and Supplemental Figure 2A). Further, CD9-MKO HFD eWAT also displayed reduced expression of the LAM markers TREM2 and OPN compared with control HFD eWAT (Supplemental Figure 2, B and C). Overall, these data indicate that myeloid-specific deletion of CD9 decreases LAM accumulation in eWAT during diet-induced obesity.

Paradoxically, flow cytometric analyses of the stromal vascular fraction revealed no difference in the frequency or number of total ATMs between CD9-MKO and control HFD mice, despite a significant reduction in CD9 expression in ATMs and other myeloid cells in CD9-MKO HFD mice (Figure 2B, Supplemental Figure 3, and Supplemental Figure 4, A–E). Likewise, the total number of CD45^+^ immune cells and the distribution of other immune cell subsets in the stromal vascular fraction were unchanged (Supplemental Figure 4, F and G).

To explain the discrepancy between our immunofluorescence and flow cytometry findings, we hypothesized that a subset of ATMs might be lost during stromal vascular fraction preparation. Prior work has shown that some ATMs can remain tightly adherent to adipocytes and therefore partition into the adipocyte fraction, which is typically discarded during flow cytometry processing (52). To assess whether CD9^+^ ATMs associate with adipocytes, we performed high-resolution imaging of eWAT from control HFD and CD9-MKO HFD mice. Consistent with our prior studies (11, 23), CD9^+^ ATMs localized to crown-like structures surrounding adipocytes, including those lacking Perilipin-1 (PLIN1) expression, a feature of dying adipocytes (Figure 2C and Supplemental Figure 5) (53). In addition, CD9-MKO HFD mice exhibited fewer F4/80^+^ crown-like structures compared with control HFD mice (Figure 2, C and D). Three-dimensional reconstruction further confirmed that CD9^+^ ATMs reside in close proximity to adipocytes and are thus appropriately positioned for direct adhesion (Figure 2E).

Since CD9^+^ ATMs were closely associated with adipocytes, we hypothesized that CD9^+^ ATMs may be found within the adipocyte fraction. Indeed, we observed that CD9^+^ F4/80^+^ ATMs could be found attached to adipocytes within the floating adipocyte fraction (Figure 2F). To test whether ATM-intrinsic CD9 influences this retention, we quantified macrophage markers in both the stromal vascular fraction and adipocyte fraction from eWAT of control HFD and CD9-MKO HFD mice. Consistent with flow cytometry data, there were minimal differences in the expression of pan-macrophage markers (*Adgre1* [F4/80] and *Cd68*) or LAM markers (*Cd9*, *Trem2*, and *Spp1* [OPN]) in the stromal vascular fraction (Figure 2G). However, all 5 markers were reduced in the eWAT adipocyte fraction from CD9-MKO HFD mice compared with control HFD mice (Figure 2H). These findings demonstrate that macrophage-intrinsic CD9 promotes ATM-adipocyte interactions and is essential for the efficient formation of crown-like structures during obesity.

### CD9 regulates integrin expression and macrophage-adipocyte adhesion.

**Figure 3:**
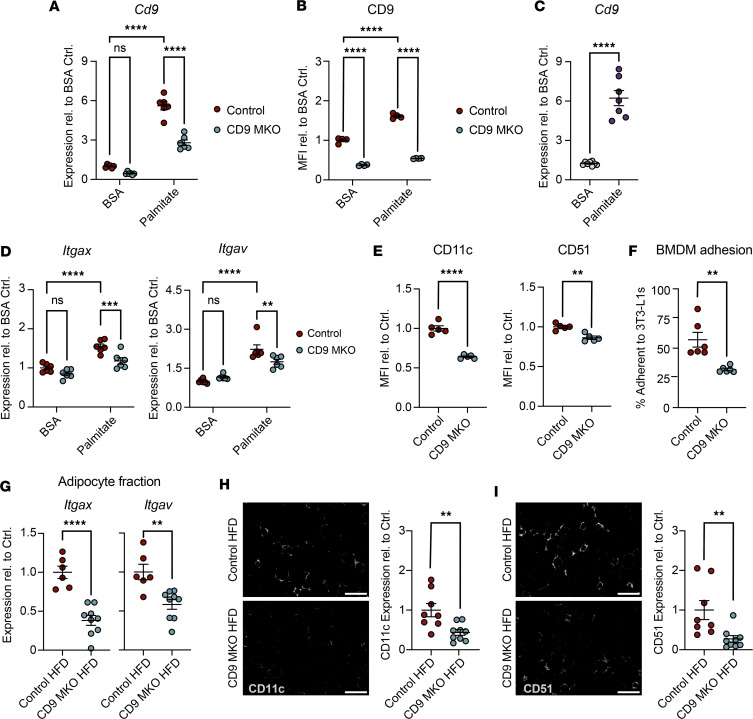
CD9 promotes macrophage integrin expression and adhesion. (**A** and **B**) BMDMs from control (*Cd9^fl/fl^*) and CD9-MKO (*Cd9^fl/fl^ LysMCre*^+/–^) mice were treated with BSA or palmitate for 24 hours. (**A**) Expression of *Cd9* was assessed by qPCR (*n* = 6). Shown as expression relative to BSA controls (Ctrl). (**B**) Surface expression of CD9 shown as relative MFI compared with BSA controls. BMDMs were gated as live CD45^+^ CD11b^+^ F4/80^+^ CD64^+^ cells (*n* = 4). (**C**) Blood monocytes isolated from WT mice fed a normal chow diet were treated with BSA or palmitate for 24 hours. Expression of *Cd9* was assessed by qPCR (*n* = 7–8). Monocytes were gated using the gating strategy in Supplemental Figure 3. (**D**) Expression of integrins *Itgax* and *Itgav* by qPCR in BMDMs from control and CD9-MKO mice treated with BSA or palmitate (*n* = 6). (**E**) Flow cytometry of CD11c (*Itgax*) and CD51 (*Itgav*) shown as MFI relative to control BMDMs treated with palmitate (*n* = 5). (**F**) Adhesion assay of control or CD9-MKO BMDMs on differentiated 3T3-L1 adipocytes (*n* = 6). (**G**) Relative expression of *Itgax* and *Itgav* in epididymal white adipose tissue (eWAT) adipocyte fraction isolated from control and CD9-MKO mice fed an HFD (*n* = 6–9). (**H** and **I**) Representative images and quantification of immunofluorescence staining of CD11c (**H**) and CD51 (**I**) in eWAT. Data shown as relative MFI (*n* = 8–9). Scale bar: 200 μm. Data shown as combined data from 2–3 independent experiments (**C**, **G–I**) or representative data from 3 independent experiments (**A**, **B**, and **D**–**F**). qPCR is shown as ΔCt relative to *Hprt* normalized to BSA controls. Data presented as mean ± SEM (**A**–**I**); 2-way ANOVA with Fisher’s LSD test (**A**, **B**, and **D**) or unpaired 2-tailed Student’s *t* test (**C**, and **E**–**I**). ns, not significant; **P < 0.01, ***P < 0.001, ****P < 0.0001.

To investigate the functional role of CD9 in macrophage-adipocyte adhesion, we utilized an in vitro model in which BMDMs were metabolically activated with palmitate. Palmitate-treated BMDMs recapitulate key transcriptional and functional features of obesity-associated ATMs (9, 20). However, it is not known whether CD9 expression is induced by this metabolic stimulus. Metabolic activation with palmitate strongly induced the expression of *Cd9* transcript and surface CD9 protein expression in BMDMs (Figure 3, A and B, and Supplemental Figure 6, A–C). CD9 deletion did not affect BMDM survival, differentiation, or proliferation of palmitate-treated BMDMs (Supplemental Figure 6, D–F). Further, palmitate also induced *Cd9* expression in sort-purified blood monocytes from WT mice fed a normal chow diet (NCD; Figure 3C). Together, these results suggest that CD9 upregulation is a general feature of monocyte and macrophage metabolic activation.

Given the established role of CD9 in regulating cell-cell adhesion, we hypothesized that CD9 may promote macrophage-adipocyte adhesion by recruiting and stabilizing integrins at the cell surface (26, 30, 31). Consistent with this idea, metabolic activation of BMDMs with palmitate increased expression of the integrins *Itgax* (CD11c) and *Itgav* (CD51) in a CD9-dependent manner (Figure 3D). Further, palmitate-treated BMDMs from CD9-MKO mice had reduced surface levels of CD11c and CD51 compared with palmitate-treated control BMDMs (Figure 3E). As it has previously been described that ATMs form tight adhesions to adipocytes via integrin interactions, we next assessed whether CD9 affects this interaction (52, 54). We found that CD9-MKO BMDMs had reduced adhesion to 3T3-L1 adipocytes compared with control BMDMs, identifying a key role for CD9 in macrophage-adipocyte interactions (Figure 3F).

Next, to determine whether CD9 deletion also alters integrin expression in vivo, we assessed *Itgax* and *Itgav* levels in eWAT of control and CD9-MKO HFD mice. Consistent with our in vitro data, deletion of CD9 reduced *Itgax and Itgav* expression in the eWAT adipocyte fraction (Figure 3G). On the other hand, *Itgax* but not *Itgav* was decreased in the eWAT stromal vascular fraction of CD9-MKO HFD mice compared with control HFD mice (Supplemental Figure 6G). Immunofluorescence staining similarly showed decreased CD11c and CD51 protein expression in CD9-MKO HFD eWAT compared with controls (Figure 3, H and I). Overall, these results suggest that CD9 regulates integrin expression in metabolically activated macrophages, both in vitro and in vivo, providing a potential mechanism by which CD9 influences macrophage-adipocyte interactions.

### Myeloid-intrinsic CD9 mediates adipose tissue remodeling during obesity.

**Figure 4:**
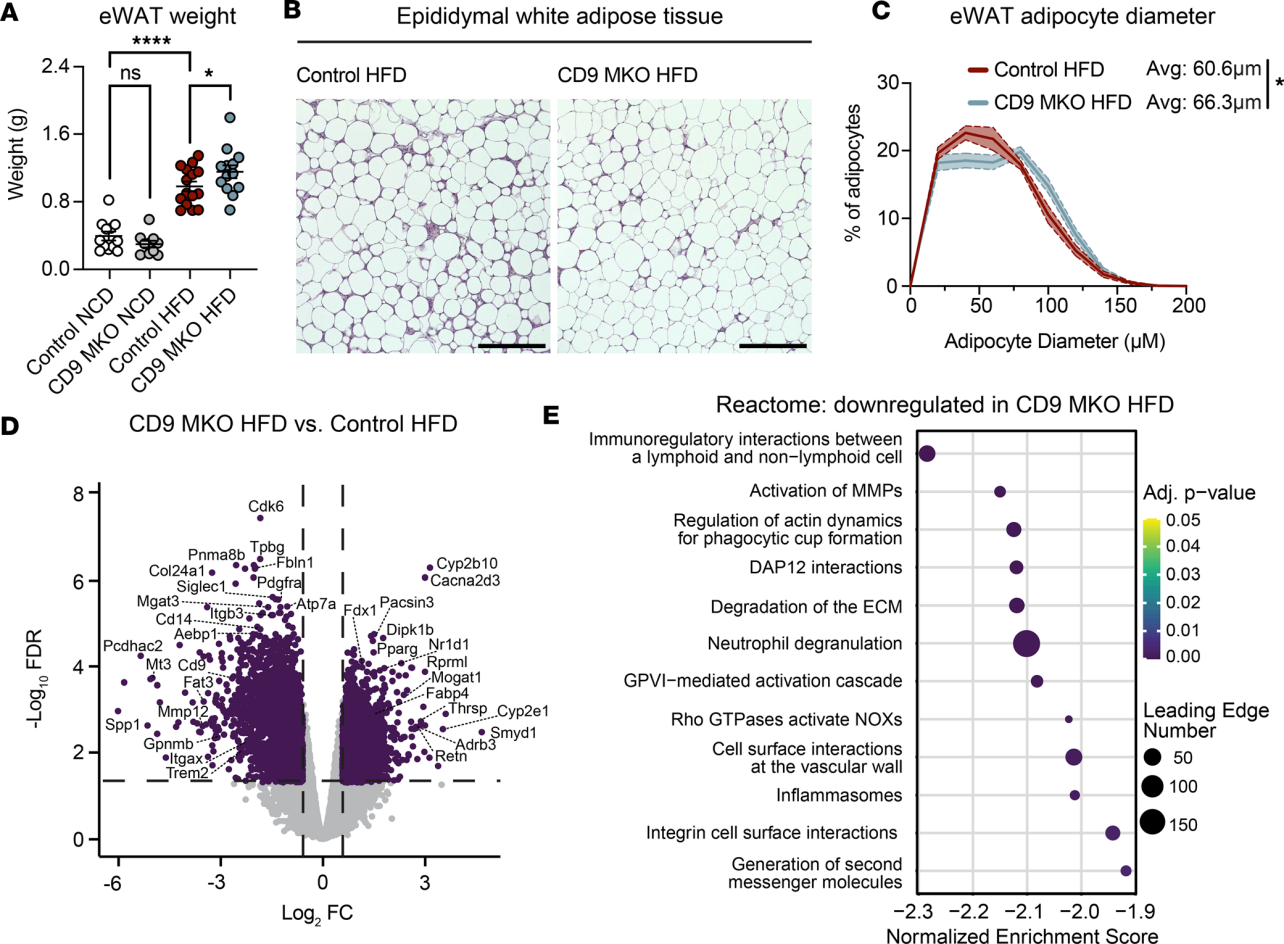
Myeloid-intrinsic CD9 alters adipose tissue remodeling during obesity. Epididymal white adipose tissue (eWAT) was collected from male control (*Cd9^fl/fl^*) and CD9-MKO (*Cd9^fl/fl^ LysMCre*^+/–^) mice fed either an NCD or HFD for 12 weeks. (**A**) eWAT mass (*n* = 10–15). (**B**) Representative H&E staining of eWAT in control or CD9-MKO HFD mice (*n* = 11–12). Scale bar: 400 μm. (**C**) Frequency distribution and mean adipocyte diameter calculated from H&E images in **B** (*n* = 11–12). (**D** and **E**) Bulk RNA-Seq was performed on eWAT depots from control and CD9-MKO mice (*n* = 4). (**D**) Volcano plot showing the log_2_ fold-change (FC; *x* axis) and adjusted *P* value (–log_10_ FDR; *y* axis) of genes between CD9-MKO HFD and control HFD groups. Significantly differentially expressed genes (log_2_ FC > 0.58 and FDR < 0.05) are shown in purple. (**E**) Pathway enrichment analysis showing the most significant (by normalized enrichment score) nonredundant pathways downregulated in eWAT. Pooled data from 3 independent experiments (**A**–**C**) or data from 1 experiment (**D** and **E**). Data presented as mean ± SEM (**A** and **C**); 1-way ANOVA with Fisher’s LSD test (**A**) or unpaired 2-tailed Student’s *t* test between means (**C**). ns, not significant; *P < 0.05, ****P < 0.0001. Avg, average; MMP, matrix metalloproteinases; NOXs, NADPH oxidases.

Given that ATMs are known to have both beneficial and pathological effects on adipose tissue adaptation in obesity, we next assessed how myeloid-specific CD9 deletion influences adipose tissue remodeling during obesity. To investigate this, we examined eWAT from CD9-MKO and control mice fed either an NCD or an HFD for 12 weeks. CD9-MKO mice exhibited slightly increased eWAT depots following HFD, but not NCD, compared with controls (Figure 4A). Histological analysis further demonstrated larger adipocyte size in CD9-MKO HFD eWAT compared with controls (Figure 4, B and C, and Supplemental Figure 7, A and B).

To further assess how loss of CD9 influences adipose tissue remodeling, we performed bulk RNA-Seq of eWAT from CD9-MKO HFD mice and control HFD mice. We identified 1,873 genes upregulated and 2,204 genes downregulated in the eWAT of CD9-MKO HFD mice compared with control HFD mice (Figure 4D). Gene set enrichment analysis revealed that the top downregulated pathways in CD9-MKO HFD eWAT were associated with inflammatory cell activation and function, ECM remodeling, and cell adhesion (Figure 4E). Consistent with our previous data, expression of *Itgax* and *Itgav* was reduced in whole eWAT from CD9-MKO HFD mice compared with control HFD mice (Supplemental Figure 7, C and D). In contrast, the top upregulated pathways included those related to cellular metabolism and mitochondrial function (Supplemental Figure 7E). Collectively, these findings demonstrate that myeloid-intrinsic CD9 broadly regulates inflammatory, adhesive, and profibrotic remodeling programs in adipose tissue during diet-induced obesity.

### Myeloid-intrinsic CD9 promotes adipose tissue fibrosis during diet-induced obesity.

**Figure 5:**
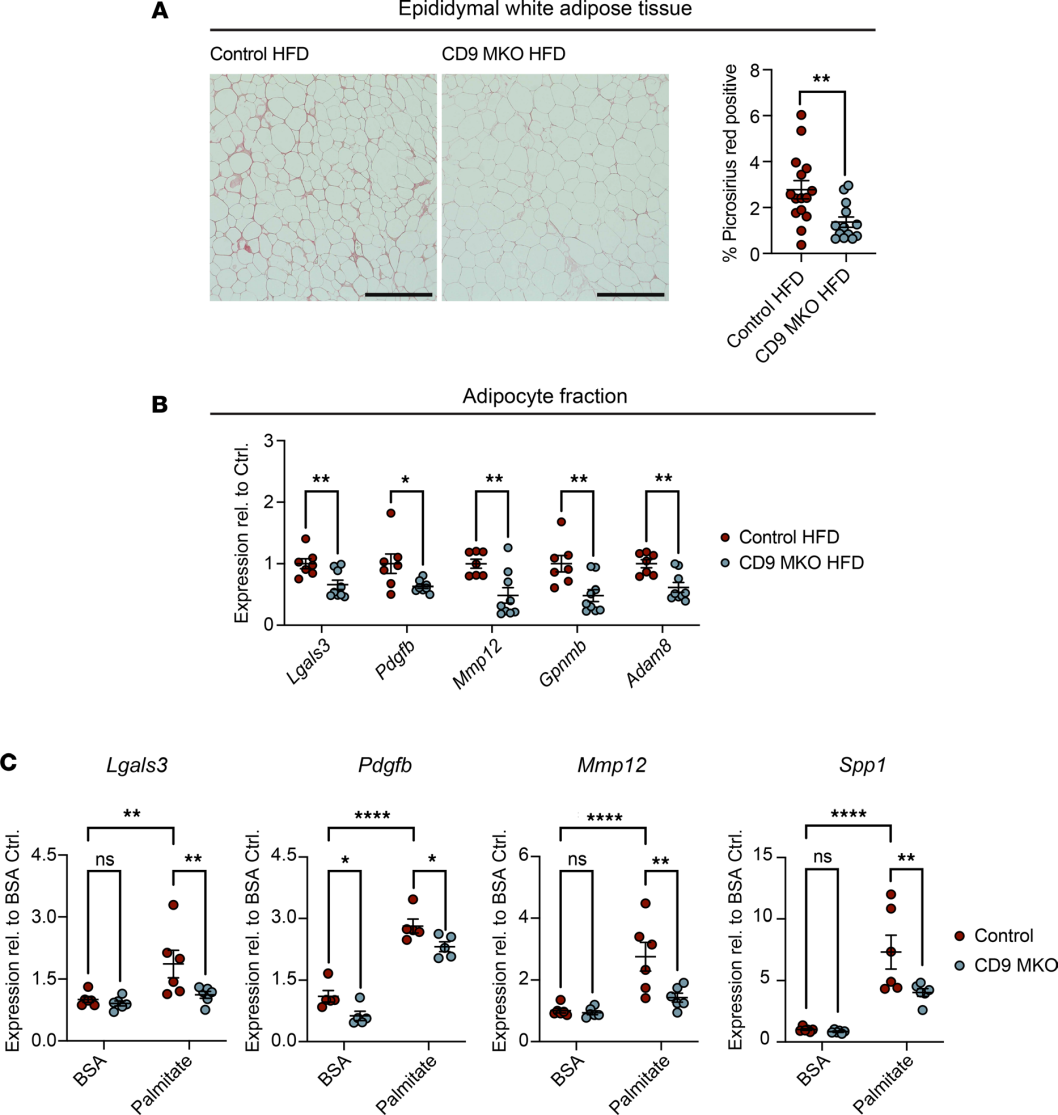
Myeloid-intrinsic CD9 promotes adipose tissue fibrosis and macrophage profibrotic gene expression during obesity. Epididymal white adipose tissue (eWAT) was collected from male control (*Cd9^fl/fl^*) and CD9-MKO (*Cd9^fl/fl^ LysMCre*^+/–^) mice fed an HFD for 12 weeks. (**A**) Representative images and quantification of Picrosirius red staining of eWAT from control HFD and CD9-MKO HFD mice (*n* = 13–15). Scale bar: 400 μm. (**B**) qPCR for fibrosis-associated genes in the adipocyte fraction isolated from control and CD9-MKO eWAT (*n* = 7–9). Data shown as ΔCt relative to a normalization factor relative to control HFD samples. (**C**) Expression of fibrosis-associated genes *Lgals3*, *Pdgfb*, *Mmp12*, and *Spp1* in BMDMs from control and CD9-MKO mice treated with BSA or palmitate for 24 hours (*n* = 5–6). Data shown as ΔCt relative to *Hprt* normalized to BSA controls. Pooled data (**A** and **B**) or representative data (**C**) from 3 independent experiments. Data presented as mean ± SEM; unpaired 2-tailed Student’s *t* test (**A** and **B**) or 2-way ANOVA with Fisher’s LSD test (**C**). ns, not significant; *P < 0.05, **P < 0.01, ****P < 0.0001.

Given that CD9^+^ ATMs were enriched for ECM remodeling genes and that these pathways were downregulated in CD9-MKO HFD eWAT compared with controls, we next investigated whether loss of myeloid-intrinsic CD9 affected eWAT fibrosis during obesity. Histological analysis demonstrated reduced collagen deposition in CD9-MKO HFD eWAT compared with controls (Figure 5A). Next, we investigated whether this reduction in fibrosis was associated with changes in the transcriptional profile of eWAT adipocyte fraction. Fibrosis-associated genes (*Lgals3*, *Pdgfb*, *Mmp12*, *Gpnmb*, and *Adam8*) were decreased in the adipocyte fraction of CD9-MKO HFD mice compared with control HFD mice (Figure 5B). Notably, with the exception of *Lgals3*, these genes have either very low or no expression in adipocytes, suggesting that the reduction in expression of these genes primarily reflects the loss of CD9^+^ ATMs adhering to adipocytes within the adipocyte fraction (55).

### CD9 regulates the expression of fibrosis-associated genes in macrophages in vitro.

LAMs are characterized by the expression of lipid metabolic, proinflammatory, and profibrotic programs (11, 23, 40). To determine whether CD9 contributes to these hallmark features, we assessed the effects of CD9 deficiency on metabolically activated macrophages. We found that CD9 did not alter the expression of lipid uptake and metabolism genes, including *Plin2*, *Cd36*, *Lpl*, and *Lipa*, in palmitate-treated BMDMs (Supplemental Figure 8A). Similarly, induction of the proinflammatory cytokines *Tnfa* and *Il6* was unaffected by CD9 deletion (Supplemental Figure 8B). Finally, loss of CD9 did not influence intracellular lipid accumulation, as assessed by boron-dipyrromethene (BODIPY) staining (Supplemental Figure 8C).

Since loss of CD9 did not affect lipid metabolism or proinflammatory genes, we next examined whether CD9 influences expression of other LAM-associated genes. Palmitate treatment induced the expression of LAM genes *Lgals3*, *Pdgfb*, *Mmp12*, and *Spp1*, but not *Trem2*, *Gpnmb*, or *Adam8*, in BMDMs (Figure 5C and Supplemental Figure 8D). Furthermore, the induction of *Lgals3*, *Pdgfb*, *Mmp12*, and *Spp1* was dependent on CD9 expression, whereas *Trem2*, *Gpnmb*, and *Adam8* expression was unaffected by CD9 deficiency. Consistent with our RNA-Seq findings, *Spp1*, *Lgals3*, *Mmp12*, and *Pdgfb* are well-established mediators of ECM remodeling and are expressed by macrophages in fibrotic disease (11, 36, 40, 41, 56–58). Together, these results suggest that myeloid-intrinsic CD9 promotes the development of adipose tissue fibrosis during obesity by modulating the expression of fibrosis-associated genes.

To further understand how CD9 regulates fibrosis-associated gene expression, we examined the contribution of integrin signaling to the expression of CD9-dependent genes. Treatment of BMDMs with blocking antibodies against CD11c or CD51 resulted in reduced expression of *Lgals3*, *Pdgfb*, and *Mmp12*, but not *Spp1*, compared with treatment with isotype controls (Supplemental Figure 9). These findings suggest that integrin-mediated binding or signaling may represent one mechanism through which CD9 modulates the expression of fibrosis-associated genes in macrophages.

### Myeloid-intrinsic CD9 worsens obesity-associated metabolic dysfunction.

**Figure 6:**
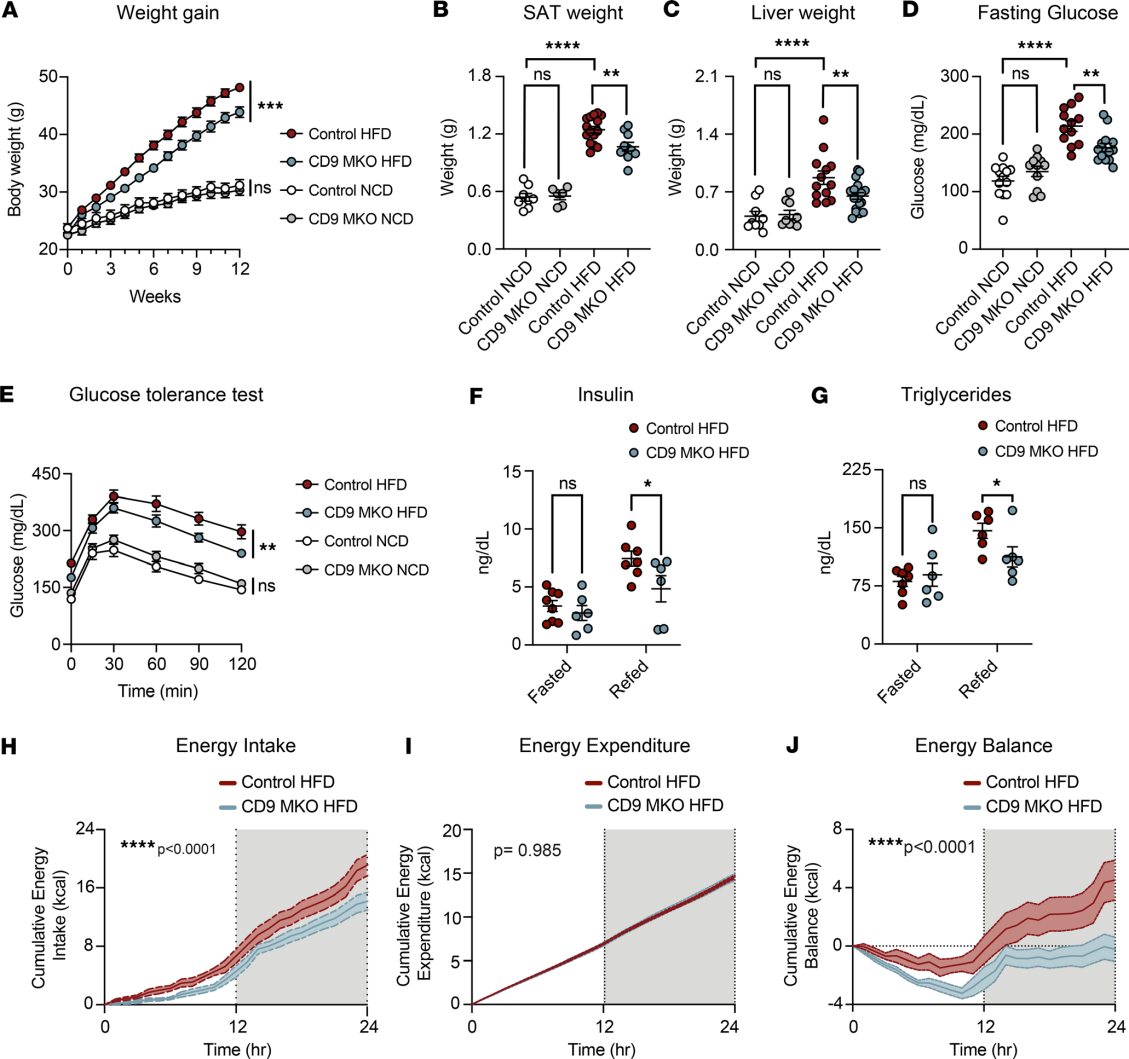
Myeloid-intrinsic CD9 contributes to obesity-associated metabolic dysfunction. (**A**) Weekly body weights of male control (*Cd9^fl/fl^*) and CD9-MKO (*Cd9^fl/fl^ LysMCre*^+/–^) mice fed either an NCD or HFD for 12 weeks (*n* = 10–29). (**B** and **C**) Tissue weights in control or CD9-MKO mice fed either an NCD or HFD for 12 weeks. (**B**) Subcutaneous (inguinal) adipose tissue weight (*n* = 6–15). (**C**) Liver (left lobe) weight (*n* = 9–18). (**D**) Glucose levels after a 16-hour fast in control or CD9-MKO mice fed an HFD for 12 weeks (*n* = 11–14). (**E**) Intraperitoneal glucose tolerance test (GTT) was performed in control or CD9-MKO mice after 12 weeks of NCD or HFD (*n* = 11–14). (**F**) Plasma insulin levels measured by ELISA after fasted (16 hours) or refed (4 hours) conditions (*n* = 6–8). (**G**) Plasma triglyceride levels after fasted (16 hours) or refed (4 hours) conditions (*n* = 6–7). (**H**–**J**) Cumulative energy intake (*n* = 6–8; **H**), energy expenditure (*n* = 7–9; **I**), and energy balance (*n* = 6–8; **J**) in control and CD9-MKO mice over a 24-hour period. Gray sections indicate dark photoperiods. Pooled data from 2–6 independent experiments (**A**–**J**). Data presented as mean ± SEM; 2-way ANOVA with Fisher’s LSD test (**A**, and **E**–**J**) performed separately for NCD and HFD groups (**A** and **E**) or 1-way ANOVA with Fisher’s LSD test (**B**–**D**). ns, not significant; *P < 0.05, **P < 0.01, ***P < 0.001, ****P < 0.0001.

Given that adipose tissue remodeling contributes to global metabolic dysfunction, we next examined how loss of myeloid-intrinsic CD9 affects systemic metabolic outcomes during diet-induced obesity. Despite having slightly larger eWAT depots, CD9-MKO mice gained less overall weight on an HFD than control mice (Figure 6A). This reduced weight gain was accompanied by a decrease in subcutaneous adipose tissue and liver mass, with no differences in muscle or brown adipose tissue weight (Figure 6, B and C, and Supplemental Figure 10, A–C). Notably, CD9-MKO HFD mice had increased perirenal adipose tissue, another form of visceral adipose tissue, relative to control HFD mice (Supplemental Figure 10D).

Additionally, CD9-MKO HFD mice exhibited reduced fasting glucose levels and improved glucose tolerance compared with control HFD mice (Figure 6, D and E). CD9-MKO HFD mice also had a reduction in fed plasma insulin and triglyceride levels compared with control HFD mice (Figure 6, F and G). On the other hand, CD9-MKO HFD mice had no differences in plasma non-esterified fatty acids or glycerol compared with control HFD mice in both the fed and fasted conditions (Supplemental Figure 10, E and F). Notably, the differences in weight gain, tissue mass, fasting glucose, and glucose intolerance were not observed under NCD conditions (Figure 6, A–E).

To investigate the mechanism underlying reduced weight gain in CD9-MKO HFD mice, we performed indirect calorimetry studies. CD9-MKO mice exhibited decreased energy intake, with no corresponding differences in energy expenditure (Figure 6, H and I, and Supplemental Figure 10, G and H). This reduction in caloric intake resulted in a lower net energy balance, likely contributing to the diminished weight gain observed in CD9-MKO mice on HFD (Figure 6J and Supplemental Figure 10I). Notably, differences in energy intake persisted after adjusting for lean mass, indicating that the reduced levels of energy intake observed in CD9-MKO mice were independent of differences in lean mass (Supplemental Figure 10J).

Finally, we assessed whether the weight and glucose homeostasis phenotypes observed in male mice were also present in female mice. In contrast to males, female CD9-MKO HFD mice showed no differences in weight gain, adipose tissue mass, or liver mass compared with control HFD mice (Supplemental Figure 11, A–D). Further, female CD9-MKO mice had no differences in gonadal white adipose tissue adipocyte size, crown-like structure formation, or fibrosis (Supplemental Figure 11, E–H). Consistent with these findings, fasting glucose levels and glucose intolerance were unchanged in female CD9-MKO HFD mice (Supplemental Figure 11, I and J). These sex-specific differences may reflect the lower frequency of CD9^+^ ATMs in female gonadal white adipose tissue, as female mice did not exhibit the prominent ATM accumulation observed in male mice (Supplemental Figure 11, K–M), in agreement with previous reports (49, 51).

## Discussion

ATMs play both beneficial and detrimental roles during obesity-associated adipose tissue remodeling. A deeper understanding of the molecules and mechanisms that drive the pathogenic activation of ATMs is critical for developing new therapeutic strategies for obesity and its metabolic complications. In this study, we demonstrate that CD9 is required for macrophage-adipocyte adhesion and the formation of crown-like structures. Additionally, loss of CD9 attenuated the profibrotic activation of ATMs and protected against pathogenic adipose tissue remodeling during diet-induced obesity. Consistent with CD9 serving a primarily pathogenic role in macrophages, myeloid-specific CD9 deletion improved systemic metabolic outcomes in obese mice. These findings establish CD9 as a critical regulator of pathogenic ATM functions, highlighting it as a potential target for mitigating obesity-associated metabolic dysfunction.

Since the initial identification of CD9 as a marker of LAMs in adipose tissue, CD9 upregulation has been observed in LAM-like macrophages across multiple murine and human tissues. This macrophage subset is characterized by the expression of lipid uptake and metabolism genes, intracellular lipid accumulation, proinflammatory cytokine production, and presence in crown-like structures (11, 23). Emerging evidence suggests that the upregulation of lipid metabolism may be an early feature of LAM activation that occurs prior to crown-like structure formation and the induction of CD9 expression (10, 41). Despite CD9’s widespread use as a defining LAM marker, it has remained unclear whether CD9 actively contributes to LAM lipid metabolic programs or is simply upregulated as a downstream consequence of their metabolic activation state.

CD9^–^ ATMs are thought to represent a transitional state between monocytes and CD9^+^ ATMs (11, 23, 41, 59). Therefore, we compared the transcriptomes of ATMs during obesity based solely on CD9 expression. Although CD9^+^ ATMs expressed higher levels of certain genes involved in lipid metabolism and uptake, we found that the loss of CD9 did not alter the expression of key lipid metabolism genes or lipid accumulation. Notably, palmitate robustly induced the expression of CD9 in BMDMs and sort-purified blood monocytes. Together, these findings suggest that CD9 does not drive LAM development but is instead upregulated as a consequence of macrophage metabolic activation. Further studies are needed to elucidate the molecules and mechanisms governing LAM development and metabolic activation, as well as the specific role of CD9 in these processes.

Crown-like structures are sites of close interactions between adipocytes and LAMs and are thought to facilitate the exchange of lipids, cytokines, and other signaling molecules (12, 32, 46, 60–63). Previous studies have demonstrated that ATMs adhere tightly to adipocytes within crown-like structures, partly through integrin-mediated interactions (52, 54). We identified CD9 as a key positive regulator of these interactions, as myeloid-specific deletion of CD9 disrupted ATM-adipocyte associations both in vitro and in vivo, leading to the loss of ATMs within the adipocyte fraction and a marked reduction in crown-like structures. Our data suggest that CD9 may contribute to this adhesive phenotype by positively regulating the expression of integrins, such as CD11c (*Itgax*) and CD51 (*Itgav*), which are critical for cell-cell and cell-ECM adhesion (31, 64, 65). Additionally, CD9 may directly facilitate cell-cell adhesion through direct interactions with partner molecules on adjacent cells (30, 66).

Importantly, our findings have methodological implications. A subset of CD9^+^ LAMs remains associated with adipocytes and partitions into the floating adipocyte fraction during standard adipose tissue processing, potentially leading to underestimation of this highly adherent ATM subset when only the stromal vascular fraction is analyzed. Conversely, as previously noted, adipocyte fractions may contain contaminating adherent ATMs (52), underscoring the need for careful interpretation of studies utilizing either fraction.

There is evidence that ATMs play important protective functions during adipose tissue remodeling through the phagocytosis and clearance of dead and dying adipocytes (10, 11). However, they also contribute to adipose tissue dysfunction through the secretion of proinflammatory and profibrotic mediators (10, 15–19). Although the specific role of CD9^+^ LAMs in adipose tissue fibrosis remains incompletely understood, CD9^+^ macrophages in the liver (SAMs) have been implicated in fibrosis promotion during steatohepatitis (36, 39, 40, 67). We found that CD9^+^ ATMs express genes involved in ECM remodeling, including those involved in both ECM synthesis and degradation. Metabolic activation with palmitate further induced the expression of fibrosis-associated genes such as *Spp1*, *Lgals3*, *Pdgfb*, and *Mmp12* in a CD9-dependent manner. On the other hand, we did not observe an upregulation of other LAM/SAM markers, including *Trem2* and *Gpnmb*. Prior work has demonstrated that type 3 cytokines can induce CD9^+^ macrophages that express high levels of *Spp1*, *Gpnmb*, *Trem2*, and *Fabp5* (40). This suggests that additional signals, such as type 3 cytokines, may be required to drive the development of CD9^+^ LAMs in vivo. Moreover, our data suggests that integrin-mediated binding and signaling may contribute to the expression of a subset of fibrosis-associated genes, highlighting a potential mechanistic link between CD9’s role in modulating integrin function and its regulation of profibrotic transcriptional programs. Future work is needed to address the mechanisms by which CD9 modulates fibrosis-associated gene expression and effector molecules during metabolic activation.

Adipose tissue remodeling is an adaptive response to excess caloric intake. However, prolonged exposure to surplus nutrients can lead to dysregulated ECM deposition and metabolic dysfunction (1, 68, 69). Adipose tissue hypoxia and inflammatory infiltration contribute to pathogenic ECM accumulation and remodeling (16, 17, 68, 70). Macrophages can exert both profibrogenic and fibrolytic functions in tissue fibrosis (15–19, 71, 72). Our study suggests that CD9^+^ LAMs are primarily profibrogenic, despite expressing genes involved in both ECM formation and degradation. CD9^+^ LAMs may contribute to fibrosis through multiple mechanisms, including promoting the proliferation and activation of fibrogenic mesenchymal stromal cells via secretion of proinflammatory cytokines such as TNF-α and IL-6, as well as CD9-dependent factors such as PDGFβ and Spp1 (OPN) (19, 56, 73). Additionally, CD9^+^ LAMs may directly contribute to ECM deposition, as observed in other fibrotic diseases, suggested by the expression of ECM components in our RNA-Seq analysis of sorted CD9^+^ ATMs (74, 75). Indeed, interstitial tissue fibrosis colocalizes with crown-like structures during obesity, supporting the idea that these sites are enriched for profibrotic ATMs (16). Thus, the reduced eWAT fibrosis observed in CD9-MKO mice may reflect both diminished profibrotic activation and impaired localization of LAMs to crown-like structures.

Myeloid-specific deletion of CD9 improved multiple obesity-associated outcomes, including insulin resistance, hyperlipidemia, and ectopic lipid deposition. Notably, these effects were sex-specific, as male mice exhibited worse metabolic outcomes, whereas female mice were largely unaffected. Female mice are known to be more resistant to obesity-associated sequelae, such as insulin resistance and adipose tissue inflammation (49, 50). This suggests that CD9^+^ LAM accumulation may play a less prominent role in adipose tissue remodeling in female mice, at least at the time point we assessed.

Our findings demonstrate that myeloid-intrinsic CD9 contributes to eWAT dysfunction, a central driver of obesity-associated metabolic impairments (1, 68, 69). Given the conserved presence of CD9^+^ macrophages across tissues, it is plausible that CD9 similarly regulates macrophage activation in other organs, such as the liver. Future studies are needed to define the role of CD9 in macrophage biology across different tissue environments during obesity. Importantly, *LysMCre* yielded only partial deletion of CD9 in macrophages, raising the possibility that more complete CD9 ablation could produce even more pronounced effects on adipose remodeling and metabolic outcomes. In addition, because *LysMCre*-mediated deletion reduced CD9 expression not only in macrophages but also in monocytes and neutrophils, CD9 may still influence additional myeloid cell populations during obesity. Further investigation is therefore warranted to determine the broader contribution of CD9 to myeloid cell function in metabolic disease.

Therapeutic targeting of ATMs has shown limited success, likely because ATMs play both beneficial and pathological roles during obesity-associated adipose tissue remodeling. In this study, we found that CD9 selectively mediates the pathogenic features of LAMs, driving the formation of crown-like structures and promoting profibrotic activation while preserving beneficial lipid metabolic functions. Moreover, myeloid-specific CD9 deletion improved adipose tissue health and mitigated obesity-associated metabolic complications. Together, these findings identify CD9 as a promising therapeutic target with the potential to modulate pathogenic ATM activity while preserving essential homeostatic functions.

## Methods

### Sex as a biological variable.

It is known that female mice are protected from adipose tissue inflammation and the development of obesity-associated sequelae. Therefore, the majority of experiments were performed using male mice. Metabolic and inflammatory phenotyping of female mice is shown in Supplemental Figure 11.

### Mice.

Mice were maintained under specific pathogen–free conditions in an animal facility at the Children’s Hospital of Philadelphia (CHOP), which has been accredited by the American Association for the Accreditation of Laboratory Animal Care. All studies were performed according to protocols the IACUC approved (24-001324). Experiments were performed using sex- and age-matched littermate controls. *Cd9^fl/fl^* mice were generated by our lab as described below. C57BL/6J (JAX stock 000664), *LysMCre* (B6.129P2-*Lyz2^tm1(cre)Ifo^*/J; JAX stock 004781), and *Vav-iCre* (B6.Cg-*Commd10^Tg(Vav1-icre)A2Kio^*/J; JAX stock 008610) mice were purchased from The Jackson Laboratory and subsequently bred in-house. All experimental mice were used between 10 and 18 weeks of age.

### Generation of Cd9^fl/fl^ mouse line.

The *Cd9^fl/fl^* mouse line was generated on a C57BL/6J background by the University of Pennsylvania CRISPR-Cas9 Mouse Targeting Core. Sanger sequencing confirmed the insertion of *LoxP* sites. Mice were backcrossed to C57BL/6J at least 6 times before further breeding. *Cd9^fl/fl^* mice were then crossed to *LysMCre* mice (B6.129P2-*Lyz2^tm1(cre)Ifo^*). *Cd9^fl/fl^* mice were genotyped using the following primers: F: CGGTAGCCCTTGTGATTTA; R: CAAACTCGGCAGGCAAGAC. The *Cd9^fl/fl^* mouse line is available from David Hill’s lab upon request.

### Diet studies.

Mice were provided with ad libitum access to water and the indicated diet. An NCD (LabDiet, 5015) was provided to all mice until 6 weeks of age. For HFD studies, mice were switched to an irradiated diet (60 kcal% fat, Research Diets, D12492) from 6 to 18 weeks of age.

### Tissue collection.

Mice were euthanized by carbon dioxide inhalation followed by cervical dislocation. Transcardiac perfusion was performed with 10 mL of PBS, and tissues were excised and weighed. Tissues used for RNA-Seq were immediately flash-frozen in liquid nitrogen and stored at –80°C. Adipose tissue used for other purposes was collected in complete media (DMEM with 10% FBS, 10 mM HEPES, 1 mM sodium pyruvate, and penicillin/streptomycin) at 37°C.

### Adipose tissue digestion.

Adipose tissue was minced and digested for 40 minutes at 37°C with end-over-end rotation in 1 mg/mL collagenase type IV (Gibco, 17104019) and 0.1 mg/mL DNase I grade II (Roche, 10104159001) in complete media. Digested tissue was passed through a 100 μM cell strainer (CELLTREAT, 229485). After filtration, samples were centrifuged for 5 minutes at 450*g*. The adipocyte fraction was removed using a 1 mL pipette, and the remainder of the sample was used for isolation of the stromal vascular fraction.

The adipocyte fraction was washed with PBS 3 times. Following each wash, adipocyte fraction samples were left upright for 10 minutes to allow adipocytes to float. The floating fraction was collected with a 1 mL pipette and transferred to a new tube. The adipocyte fraction was then frozen in TRIzol (Invitrogen, 15596018) for qPCR or further processed for immunofluorescence imaging.

To isolate the stromal vascular fraction, samples were centrifuged at 450*g* for 5 minutes and washed with PBS. Red blood cells were lysed using ACK lysing buffer (Quality Biological, 118-156-721EA). Samples were frozen in TRIzol for qPCR or used for flow cytometry experiments described below.

### Flow cytometry and fluorescence-activated cell sorting.

The stromal vascular fraction was isolated as detailed above. Cell number and viability were quantified using trypan blue (Gibco, 15-250-061) staining and a TC20 Automated Cell Counter (Bio-Rad). Isolated cells were blocked and stained using fluorophore-conjugated antibodies, live-dead discrimination dyes, and lipid dyes listed in Supplemental Table 1. Single-stain controls and fluorescence-minus-one controls were used as necessary. ATMs for bulk RNA-Seq were sorted using a FACS Aria II (BD Biosciences). ATMs and blood monocytes for qPCR were sorted using an Aurora CS (Cytek). ATMs were cultured in complete media for 36 hours before being frozen in TRIzol for qPCR. Monocytes were cultured in complete media with BSA or palmitate for 24 hours before being frozen in TRIzol. Flow data were collected using an Aurora flow cytometer (Cytek) and analyzed using FlowJo v10 (BD Biosciences). Immune cells were gated as previously described (23, 32).

### Immunofluorescence of adipocyte fraction samples.

Adipocyte fractions were isolated as detailed above. The adipocyte fraction was fixed using 4% paraformaldehyde (FD Neurotechnologies, PF101) for 15 minutes at room temperature. The adipocyte fraction was then allowed to float for 10 minutes, collected into a new tube, and washed once with PBS. Cells were then blocked with CD16/32 (1:200) for 10 minutes in PBS. After blocking, cells were stained with Hoechst 33342 (Thermo Fisher Scientific, 66249, 1:250), BODIPY 493/503 (Invitrogen 1:1,000), F4/80 (APC BD Biosciences, 1:500), and CD9 (PE BioLegend, 1:500) at room temperature for 30 minutes in the dark. Antibody information is listed in Supplemental Table 1. Samples were washed 3 additional times with PBS before imaging. Adipocyte fraction samples were imaged using a Zeiss Observer Widefield Microscope. Image analysis was conducted in Fiji.

### BMDM studies.

BM was collected from male mice between 10 and 15 weeks of age. BM was isolated by flushing murine femurs and tibias with cold PBS or by crushing with a mortar and pestle. Red blood cells were lysed with ACK lysis buffer, and samples were filtered through a 70 μm cell strainer (MTC Bio, C4070). BMDMs were generated by culturing BM with 10 ng/mL of M-CSF (PeproTech, 315-03) in complete media for 7 days in 10 cm non-tissue treated polystyrene plates (VWR, 89038-968). Additional media containing M-CSF was added on day 4.

Differentiated cells were harvested by scraping with ice-cold PBS. For metabolic activation studies, BMDMs were cultured with 300 μM BSA-conjugated palmitate or BSA for 24 hours. BSA-conjugated palmitate was prepared as follows: solutions of BSA (0.34 mM, Ultra Fatty Acid-Free BSA, Roche, 03117405001) and the sodium salts of palmitate (2 mM, Sigma-Aldrich, P9767-5G) were prepared in 150 mM NaCl in water. BSA and palmitate were combined 1:1 (v/v) and allowed to conjugate for 1 hour at 37°C with gentle stirring. The pH was adjusted to approximately 7.4, and aliquots were frozen in glass vials. The final concentrations were 1 mM palmitate/0.17 mM BSA, or a 6:1 molar ratio of palmitate/BSA.

For experiments with integrin blockade, BMDMs were treated with antibodies against integrins or isotype controls at the time of treatment with BSA or palmitate. BMDMs were then cultured for 24 hours prior to harvesting for qPCR. To assess the role of CD11c in BMDM gene expression, cells were treated with 1:100 anti-CD11c (Bio X Cell, BE0038, clone N418) or polyclonal Armenian hamster IgG isotype control (Bio X Cell, BE0091). To assess the role of CD51 in BMDM gene expression, cells were treated with 1:100 anti-CD51 (BioLegend, 104102, clone RMV-7) or rat IgG1κ isotype control (BioLegend, 400401).

### 3T3-L1 BMDM adhesion assays.

First, 3T3-L1 cells (ATTC, CL-173) were grown in 24-well tissue culture-treated plates (CELLTREAT, 229124). Cells were plated at a concentration of 2 × 10^4^ cells per well and grown until they reached 100% confluency. Cells were incubated for an additional 48 hours before adding induction media (DMEM with 10% FBS, penicillin/streptomycin, 500 μM 3-isobutyl-1-methylxanthine, 1 μM dexamethasone, and 3 μg/mL insulin). After 48 hours, induction media was replaced with adipocyte maintenance media (DMEM with 10% FBS, penicillin/streptomycin, 3 μg/mL insulin). Maintenance media was replaced every 2–3 days until adipocytes were fully differentiated (8–12 days following the addition of induction media).

BMDMs were harvested by incubation with ice-cold PBS followed by gentle scraping. BMDMs were then incubated with 5 μM CFSE (Thermo Fisher Scientific, 50-591-407) for 15 minutes at 37°C and washed with PBS. Next, 1 × 10^5^ BMDMs were added to a 24-well plate containing differentiated 3T3-L1 adipocytes in complete media. Cells were incubated at 37°C for 30 minutes to allow BMDMs to adhere. Media was then removed and wells were washed 3 times with PBS. For each group, a subset of wells was not washed, and these samples were used as unwashed controls. Wells were then imaged using an EVOS FL auto microscope (Thermo Fisher Scientific). CFSE^+^ cells were counted using CellProfiler (Broad Institute). The number of CFSE^+^ cells was then divided by the average number of CFSE^+^ cells in the unwashed controls of the same genotype to calculate the percentage of adherent cells.

### Histology.

eWAT samples were fixed overnight in 4% buffered paraformaldehyde solution at 4°C, followed by ethanol dehydration. Dehydrated samples were sent to the University of Pennsylvania Veterinary School Comparative Pathology Core to perform H&E and Picrosirius red staining. Histological images were acquired using the EVOS FL auto microscope (Thermo Fisher Scientific). Adipocyte size was measured using the Adiposoft plugin in the Fiji application. Picrosirius red staining was quantified using Fiji. For female mice, crown-like structures were quantified manually in blinded H&E images. Histological analyses were performed on 3–5 images per biological replicate.

Immunofluorescence staining was performed using the following primary antibodies: anti-F4/80 (Cell Signaling Technology, 70076), anti-CD68 (Cell Signaling Technology, 97778), anti-CD11c (Cell Signaling Technology, 97585), anti-CD51 (Abcam, ab179475), anti-TREM2 (Abcam, ab305103), anti-OPN/SPP1 (Cell Signaling Technology, 88742), anti-CD9 (Abcam, ab307085), anti-Perilipin-1 (Cell Signaling Technology, 3470), and DAPI (Quanterix, FP1490). Staining was performed using the BOND RXm (Leica, DS9800). Immunofluorescence images were acquired on the EVOS FL auto microscope or Zeiss Observer 7. In male mice, crown-like structures were quantified manually in blinded F4/80 immunofluorescence images. Analyses were performed on 5 images per biological replicate.

For high-resolution imaging and 3D reconstruction, slides were imaged on a Leica TCS SP8 confocal microscope using a 63× oil immersion objective (NA = 1.4). Images were acquired at 1,024 × 1,024 pixels as *Z*-stacks (30–40 steps of 0.12 μm per field of view). Obtained images were deconvoluted using Leica Lightning Deconvolution software. Representative images represent a single plane, with brightness and contrast adjusted equivalently across samples in ImageJ (NIH). All fluorescent staining (blue, green, red, and far-red channels) were acquired using HyD detectors in the standard mode with 100% gain, and 3D image reconstruction was performed with Imaris software (v8.1.2, Oxford Instruments). Adipocyte, macrophage, and CD9 surfaces were created using the Surfaces tool with automatic settings and thresholding based on the fluorescent signals from the anti-PLIN1, anti-F4/80, or anti-CD9 antibodies, respectively.

### Blood glucose measurements.

For fed and fasting blood glucose, blood was collected via the tail vein. Glucose was measured using blood glucose strips and a BG1000 glucose monitor (Clarity Diagnostics) under fed or fasting (~16 hours) conditions. For intraperitoneal glucose tolerance tests, mice were fasted overnight (~16 hours) in a clean cage with ALPHA-Dri bedding (Lab Supply). Mice were weighed and given an intraperitoneal injection of glucose solution at a dose of 1 gram per kg body weight. Blood glucose levels were measured before (0 minutes) and after (15, 30, 60, 90, and 120 minutes) glucose administration.

### Metabolic plasma studies.

Blood was collected via tail vein under fasting (~16 hours) and refed (4 hours) conditions. Samples were collected in Microvette CB 300 lithium heparin tubes (Sarstedt) on ice. Samples were centrifuged at 13,000*g* for 10 minutes at 4°C. Plasma was removed and frozen at –80°C until further analysis. Serum insulin levels were measured using the Ultrasensitive Mouse Insulin ELISA kit (Crystal Chem, 90080) on an Epoch Spectrophotometer (Agilent BioTek). Plasma triglycerides were measured using the Infinity Triglyceride Reagent (Thermo Fisher Scientific, TR22421) on an Epoch Spectrophotometer (Agilent BioTek). Plasma non-esterified fatty acids were measured using a Fujifilm HR Series NEFA-HR(2) (999-34691, 995-34791, 999-34891, 999-35191). Plasma glycerol was measured using a free glycerol assay kit (Cell Biolabs, STA-398).

### Indirect calorimetry.

Metabolic cage experiments were performed with a Promethion CORE metabolic cage system (Sable Systems International). Mice were acclimated in the Promethion metabolic cages at 22°C for 48 hours before the recording period. Oxygen consumption (VO_2_) and carbon dioxide production (VCO_2_) were measured by indirect calorimetry every 5 minutes and used to calculate energy expenditure using the Weir equation (energy expenditure = 3.941 kcal/L × VO_2_ + 1.106 kcal/L × VCO_2_) (76). Food intake, water intake, and body mass were measured in the cages gravimetrically. Lean body mass was measured using an EchoMRI. Data were analyzed in CalR2 (https://calrapp.org/) (77). ANCOVA testing was performed in CalR2, using pretreatment lean mass as a covariate.

### RT-qPCR.

RNA was isolated using TRIzol (Invitrogen, 15596018) according to the manufacturer’s instructions. Reverse transcription was performed using the Verso cDNA Synthesis kits (Thermo Fisher Scientific, AB1453B) according to the manufacturer’s instructions. qPCR was performed using PowerUp SYBR Green Master Mix on a QuantStudio 7K Real-Time PCR System (Applied Biosystems, A25742). Primer sequences are listed in Supplemental Table 2. All reactions were run in duplicate or triplicate. For BMDM experiments, gene expression was calculated as 2^–(Ct^_gene_^–Ct^*_Hprt_*^)^. For analyses of sorted ATMs, stromal vascular fraction, and adipocyte fractions, each sample’s normalization factor (NF) was calculated as the geometric mean of the Ct values for the genes *Hprt*, *Rpl13a*, *Sdha*, *Actb*, and *B2m*. Gene expression was calculated as 2^–^(^Ct^_gene_^–NF^).

### RNA-Seq.

For bulk RNA-Seq of CD9^+^ and CD9^–^ ATMs, libraries were prepared from sort-purified cell populations as previously described (23). Briefly, SMARTer Stranded RNA-Seq kit (Clontech) and TruSeq Stranded mRNA Library prep kits (Illumina, 20020594) were used according to the manufacturer’s instructions. RNA-Seq libraries were sequenced on an Illumina HiSeq 2000.

For bulk RNA-Seq of adipose tissue, total RNA was isolated from snap-frozen tissue samples. RNA extraction was performed using the Maxwell RSC simply RNA tissue kit (Promega, AD1340). Briefly, tissue was homogenized in Thioglycerol Homogenization solution using a Maxwell RSC instrument (Promega). RNA was quantified using a NanoDrop 8000 spectrophotometer (Thermo Fisher Scientific) and an Agilent 4200 bioanalyzer system. RNA libraries were prepared using the standard Illumina protocol. RNA-Seq libraries were sequenced as paired-end, 75–100 bp read length on an Illumina NovaSeq 6000 with an SP 200 cycle kit by the CHOP Center for Applied Genomics.

Bulk RNA-Seq reads were aligned to the GRCmm39/mm10 genome using the nf-core RNA-Seq pipeline version 3.14.0 (78). In brief, FastQC was used to assess the quality of reads (79), and Trim Galore! was used for adapter and quality trimming (80). Alignment was performed with STAR version 2.7.10a (81) followed by quantification with RSEM version 1.3.1 (82). Duplicates were removed using Picard MarkDuplicates (83). Low-expression genes were filtered with the R package edgeR, followed by differential expression analysis (84). Genes were considered to have significant differential expression with an FDR less than 0.05. Gene set enrichment analysis was performed with Reactome, GO, and Wikipathways gene sets using the R package WebGestaltR with log_2_ FC as the ranking metric (85). Pathways with an adjusted *P* value of greater than 0.05 were considered significantly enriched.

### Statistics.

The method for analysis of RNA-Seq data is described above. GraphPad Prism software was used for graphing and statistical analysis. Data are presented as mean ± SEM unless otherwise stated. Comparisons between 2 groups were performed using an unpaired 2-tailed Student’s *t* test. An *F* test was used to compare the variance of the tested groups; groups with significantly different variances were compared using Welch’s *t* test. Comparisons between 3 or more groups were performed by 1- or 2-way ANOVA with Fisher’s LSD test for multiple comparisons. Outliers were identified by ROUT with Q = 1%. A *P* value of less than 0.05 was considered significant.

### Study approval.

Animal experiments were performed in accordance with ethical regulations and protocols approved by the IACUCs of CHOP and the Perelman School of Medicine at the University of Pennsylvania.

### Data availability.

Raw bulk RNA-Seq data are available in NCBI’s Gene Expression Omnibus (GEO GSE113594 and GSE311351). Raw data and values for all data points in graphs are reported in the Supporting Data Values File.

## Author contributions

Conceptualization and design of the research study were performed by JC, SJM, MAL, and DAH. Experiments and data acquisition were conducted by JC, ND, DVM, SJM, KMS, DP, RLC, and JM. Data analysis was performed by JC, ND, and DVM. Data curation and visualization were performed by JC. Writing and preparation of the original draft were performed by JC and DAH. Project administration was performed by JC, ND, and DAH. Funding was acquired by RJ, DMT, MAL, and DAH. All authors have reviewed and approved the final manuscript.

## Funding support

This work is the result of NIH funding, in whole or in part, and is subject to the NIH Public Access Policy. Through acceptance of this federal funding, the NIH has been given a right to make the work publicly available in PubMed Central.

- NIH grants K08 DK116668 and R01 HL162715 (to DAH).
- Penn Diabetes Research Center (P30DK019525) pilot grant (to DAH).
- American College of Allergy, Asthma, and Immunology Junior Faculty grant (to DAH).
- CHOP Research Institute (to DAH).
- University of Pennsylvania Theo Wilson Graduate Research Fellowship (to JC).
- NIH grant 5T32 DK007314-43 (to JC).
- American Heart Association Predoctoral Fellowship 24PRE1191220 (to JC).
- NIH grant R01 DK49780 (to MAL).
- The JPB Foundation (to MAL).
- NIH grant T32 GM007170 (to JC and SJM).
- University of Pennsylvania Michael Brown Graduate Research Fellowship (to SJM).
- NIH grant R35HL166663 (to RJ).
- Penn Rodent Metabolic Phenotyping Core (RRID: SCR_022427), supported in part by NIH grant S10-OD025098, the Cox Institute, and the Penn Institute for Diabetes, Obesity, and Metabolism.
- Penn Vet Comparative Pathology Core, supported in part by the Abramson Cancer Center Support grant (P30 CA016520) and NIH grant S10 OD023465-01A1.

## Supplementary Material

Supplemental data

Unedited blot and gel images

Supporting data values

## Version 1 02/10/2026

In-Press Preview

## Version 2 03/23/2026

Electronic publication
