# Supplementary material for: CD9 regulates macrophage-mediated remodeling of adipose tissue in obesity
Source: JCI Insight. 2026 Feb 10;11(6):e193837. doi: 10.1172/jci.insight.193837 (PMC13043082; doi:10.1172/jci.insight.193837)
Supplement: Supplemental data [file jciinsight-11-193837-s197.pdf]

Supplemental Data:

CD9 regulates macrophage-mediated remodeling of adipose tissue in obesity

Authors: Julia Chini,[1,2,3] Nicole DeMarco,[2] Dana V. Mitchell,[4] Sam J. McCright,[1,2,3] Kaitlyn M. Shen,[5] Divyansi Pandey, [1,2,3] Rachel L. Clement,[1,2,3] Jessica Miller,[2] Rajan Jain,[5] Deanne M. Taylor,[1,4] Mitchell A. Lazar,[6,7] David A. Hill[1,2,3,7] *

Affiliations:

[1]Department of Pediatrics, Perelman School of Medicine, University of Pennsylvania; Philadelphia, PA, USA.

[2]Division of Allergy and Immunology, Children's Hospital of Philadelphia; Philadelphia, PA, USA.

[3]Institute for Immunology and Immune Health, Perelman School of Medicine, University of Pennsylvania; Philadelphia, PA, USA.

[4]Department of Biomedical and Health Informatics, Children's Hospital of Philadelphia; Philadelphia, PA, USA.

[5]Departments of Medicine and Cell and Developmental Biology, Penn Cardiovascular Institute, Penn Epigenetics Institute, Perelman School of Medicine, University of Pennsylvania; Philadelphia, PA, USA

[6]Department of Medicine, Division of Endocrinology, University of Pennsylvania Perelman School of Medicine; Philadelphia, PA, USA.

[7]Institute for Diabetes, Obesity and Metabolism, Perelman School of Medicine, University of Pennsylvania; Philadelphia, PA, USA.

*Corresponding author. Email: hilld3@chop.edu. Address: Division of Allergy and Immunology, Children's Hospital of Philadelphia, Abramson Research Building, 1208B, 3615 Civic Center Blvd., Philadelphia, PA 19104. Phone: (215) 590-2549.

Chini et al.- Supplemental. Figure 1

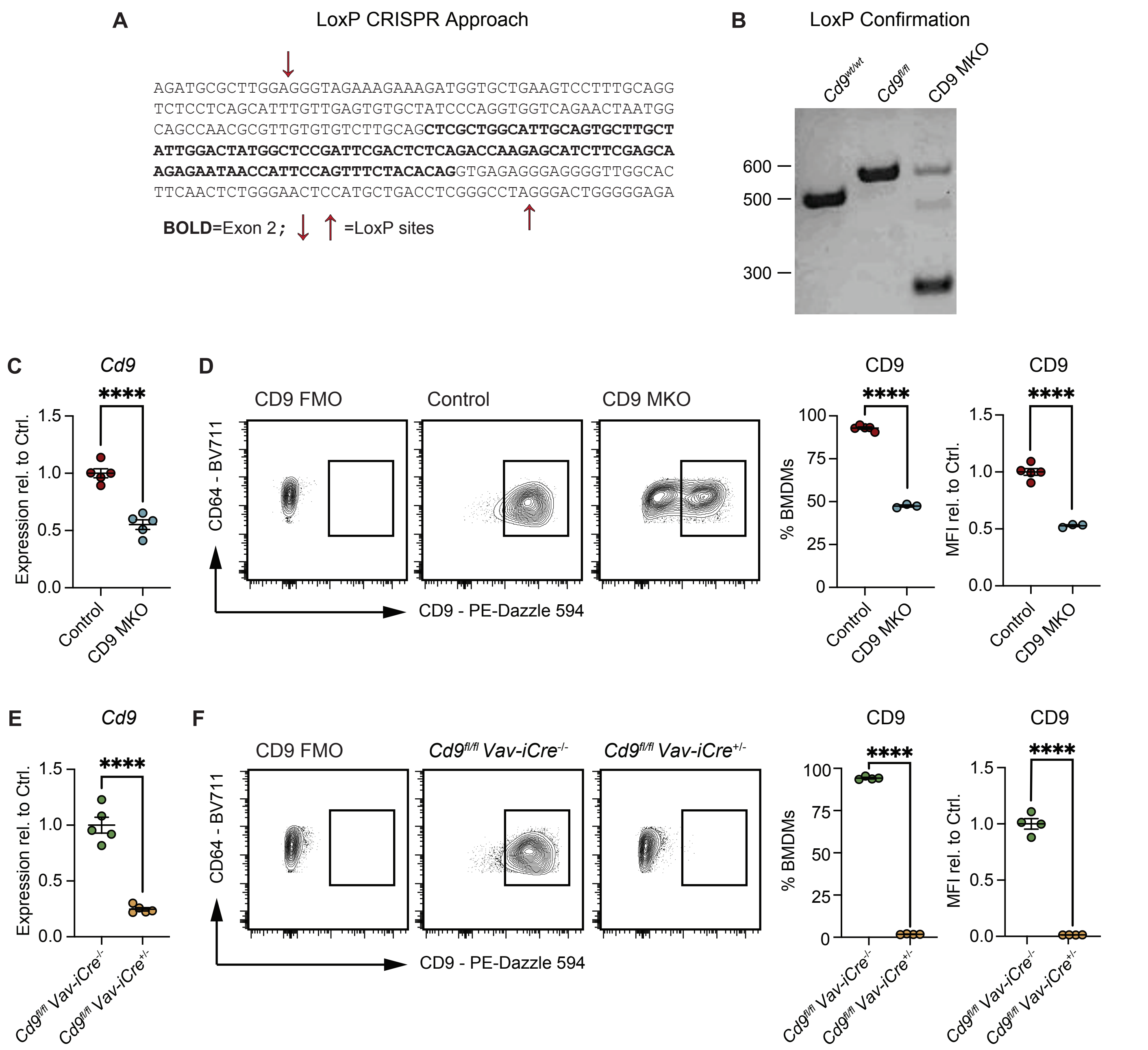

Supplementary Figure 1: Development and functional confirmation of *Cd9$^{fl/fl}$* mouse line. (**A**) The genomic sequence of murine *Cd9* showing exon 2 (bold) and surrounding intronic regions. *LoxP* sites (red arrows) were inserted using CRISPR to generate the *Cd9$^{fl/fl}$* mouse line. (**B**) Genotyping PCR gel image showing expected band sizes of the *Cd9* DNA locus from bone marrow-derived macrophages (BMDMs) isolated from wild-type, *Cd9$^{fl/fl}$*, or *Cd9$^{fl/fl}$ LysMCre$^{+/-}$* (CD9 MKO) mice. (**C**) *Cd9* expression relative to controls (Ctrl.) in unpolarized BMDMs isolated from Control (*Cd9$^{fl/fl}$*) or CD9 MKO (*Cd9$^{fl/fl}$ LysMCre$^{+/-}$*) mice (n=5). (**D**) Representative flow cytometric plots and quantification of frequency of CD9^{+} cells and mean fluorescence intensity (MFI) relative to controls in unpolarized BMDMs isolated from Control or CD9 MKO mice (n=3-5). (**E**) *Cd9* expression in unpolarized BMDMs isolated from *Cd9$^{fl/fl}$ Vav-iCre$^{-/-}$* or *Cd9$^{fl/fl}$ Vav-iCre$^{+/-}$* mice (n=5). (**F**) Representative flow cytometric plots and quantification of frequency of CD9^{+} cells and MFI relative to Ctrl. in unpolarized BMDMs isolated from *Cd9$^{fl/fl}$ Vav-iCre$^{-/-}$* or *Cd9$^{fl/fl}$ Vav-iCre$^{+/-}$* mice (n=4). Data representative of 3 independent experiments (**B-F**). Data presented as mean ± SEM (**C-F**). Statistics: unpaired Student's t-test (**C-F**). ****$p < 0.0001$.

Chini et al.- Supplemental. Figure 2

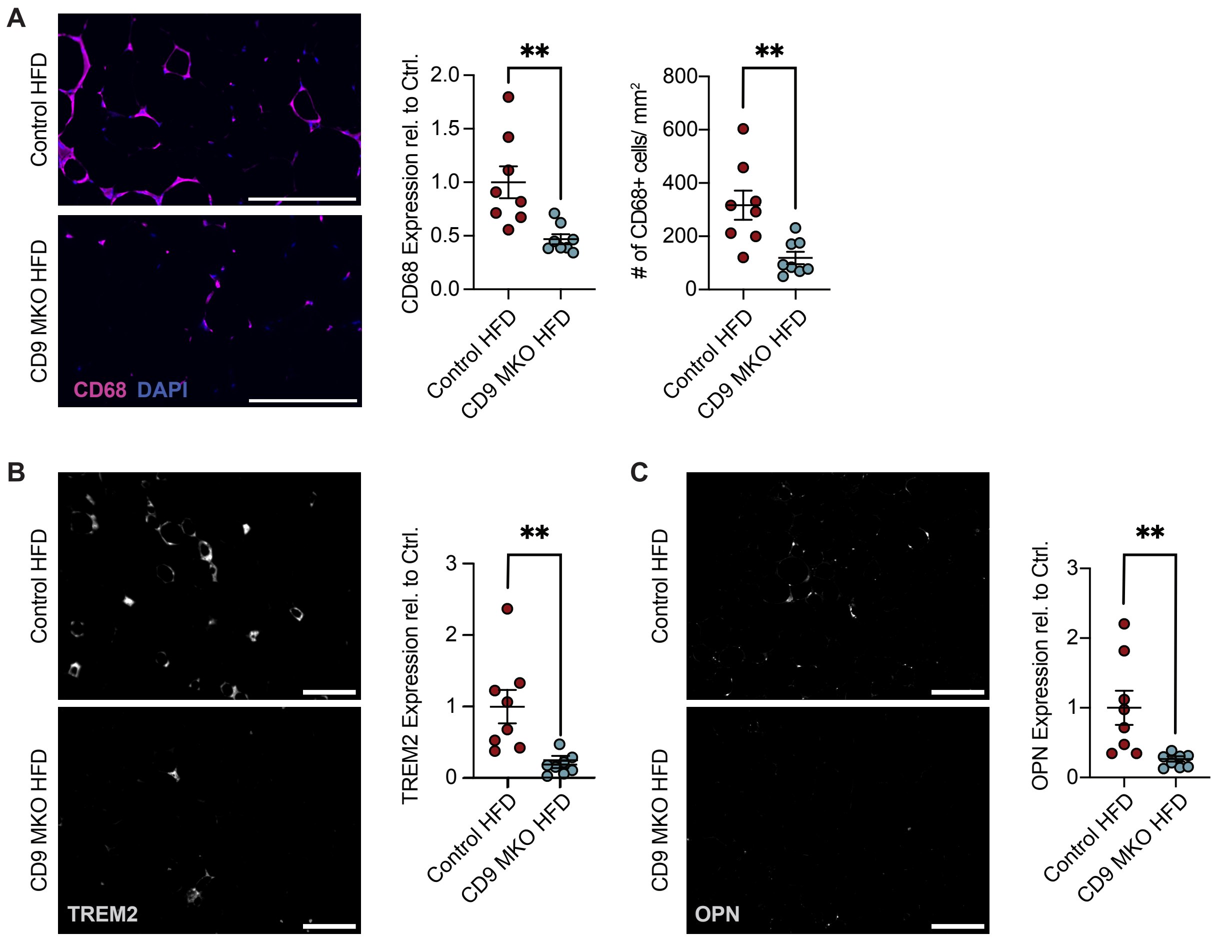

Supplementary Figure 2: CD9 regulates lipid associated macrophage accumulation in visceral adipose tissue during obesity. Analysis of epididymal white adipose tissue (eWAT) from Control (*Cd9*$^{fl/fl}$) and CD9 MKO (*Cd9*$^{fl/fl}$ *LysMCre*$^{+/-}$) male mice fed a high-fat diet (HFD) for 12 weeks. (**A**) Representative images and quantification of immunofluorescence staining of F4/80 and DAPI in eWAT tissue sections. Data shown as relative mean intensity of fluorescence of F4/80 and number of F4/80^{+} DAPI^{+} cells per field (n=8). Scale bar = 200μm. (**B** and **C**) Representative images and quantification of immunofluorescence staining of TREM2 (**B**) and OPN (**C**) in eWAT tissue sections. Data shown as relative mean intensity of fluorescence (n=8). Scale bar = 200μm. Data shown as pooled data from 2 independent experiments and presented as mean ± SEM. Statistics: unpaired Student's t-test (**A-C**). **p < 0.01.

Chini et al.- Supplemental. Figure 3

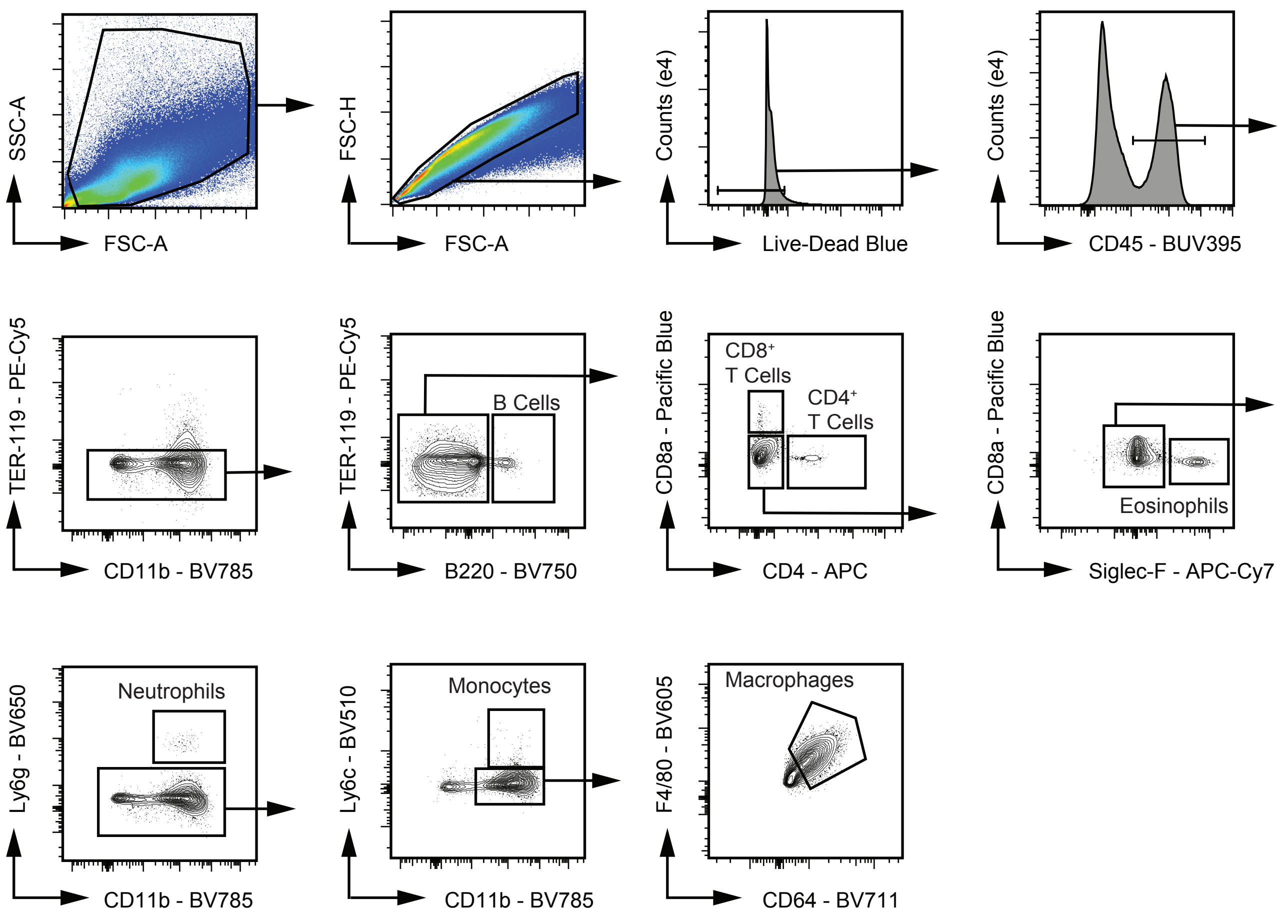

Supplementary Figure 3: Flow cytometric gating strategy of stromal vascular immune cells in eWAT. Representative flow cytometric plots of stromal vascular fraction isolated from epididymal white adipose tissue.

Chini et al.- Supplemental. Figure 4

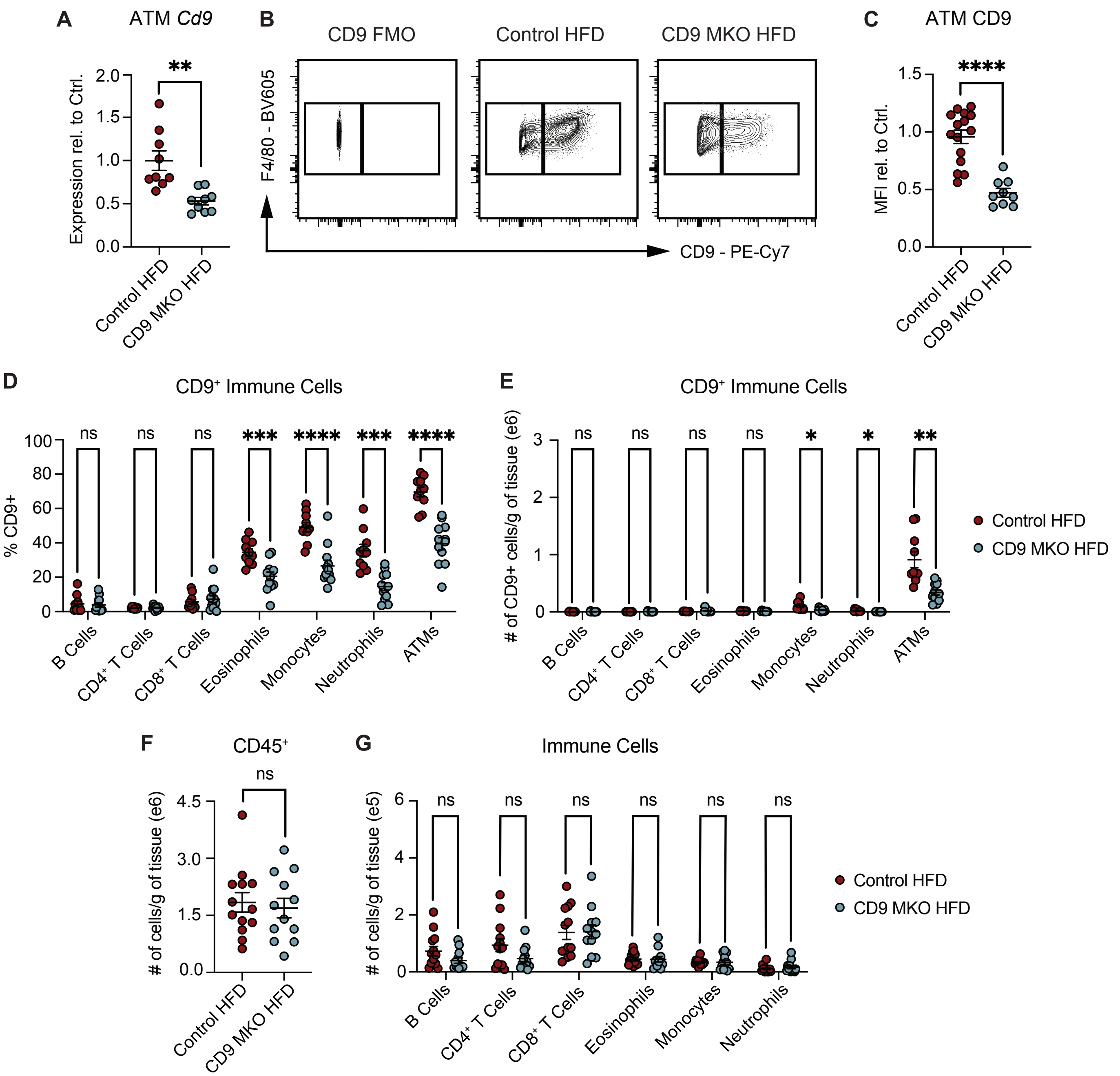

Supplementary Figure 4: Flow cytometric analysis of eWAT stromal vascular cells. (**A-G**) Analysis of epididymal white adipose tissue (eWAT) stromal vascular fraction immune cells from male Control (*Cd9*$^{fl/fl}$) or CD9 MKO (*Cd9*$^{fl/fl}$ *LysMCre*$^{+/-}$) mice fed a high fat diet (HFD) for 12 weeks. Immune cells were gated as shown in Supplementary Figure 2 (**A**) Expression of *Cd9* by qPCR in adipose tissue macrophages (ATMs) sorted from eWAT Control and CD9 MKO mice (n=9). Expression shown as ΔCt relative to a normalization factor relative to Control (Ctrl.) HFD samples. (**B** and **C**) Analysis of eWAT ATMs in Control and CD9 MKO mice by flow cytometry. (**B**) Representative flow cytometric plots of ATMs from a CD9 fluorescence minus one (FMO) control and Control or CD9 MKO mice. (**C**) CD9 Mean fluorescence intensity (MFI) presented relative to Control (n=9-15). (**D**) Frequency of CD9^{+} immune cells (n=10-12). (**E**) Number of CD9^{+} immune cells per gram of eWAT tissue (n=10-12). (**F** and **G**) Quantification of total CD45^{+} (n=12-13; **F**) and subsets of immune cells (n=12-13; **G**) by flow cytometry of Control and CD9 MKO HFD mice. Pooled data from 3 independent experiments. Data presented as mean ± SEM. Statistics: unpaired Student's t-test. ns = not significant, *p < 0.05, **p < 0.01, ***p < 0.001, ****p < 0.0001.

Chini et al.- Supplemental. Figure 5

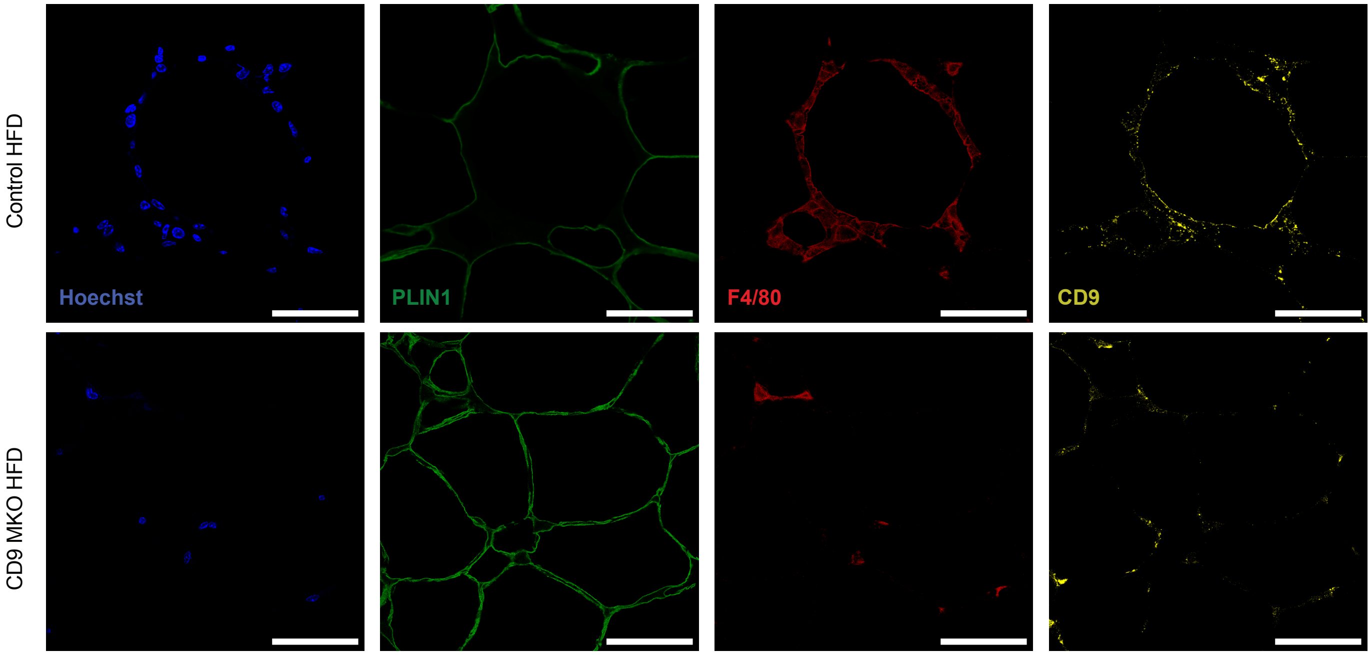

Supplementary Figure 5: High resolution imaging reveals association of CD9$^+$ ATMs with adipocytes in crown like structures. Analysis of epididymal white adipose tissue (eWAT) from Control (*Cd9*$^{fl/fl}$) and CD9 MKO (*Cd9*$^{fl/fl}$ *LysMCre*$^{+/-}$) male mice fed a high-fat diet (HFD) for 12 weeks. Representative single color high resolution images of eWAT tissue sections from Control and CD9 MKO mice shown in **Figure 2D** stained with DAPI (blue), PLIN1 (green), F4/80 (red), and CD9 (yellow) (n=6-9). Scale bar = 50μm. Data from 2 independent experiments.

Chini et al.- Supplemental. Figure 6

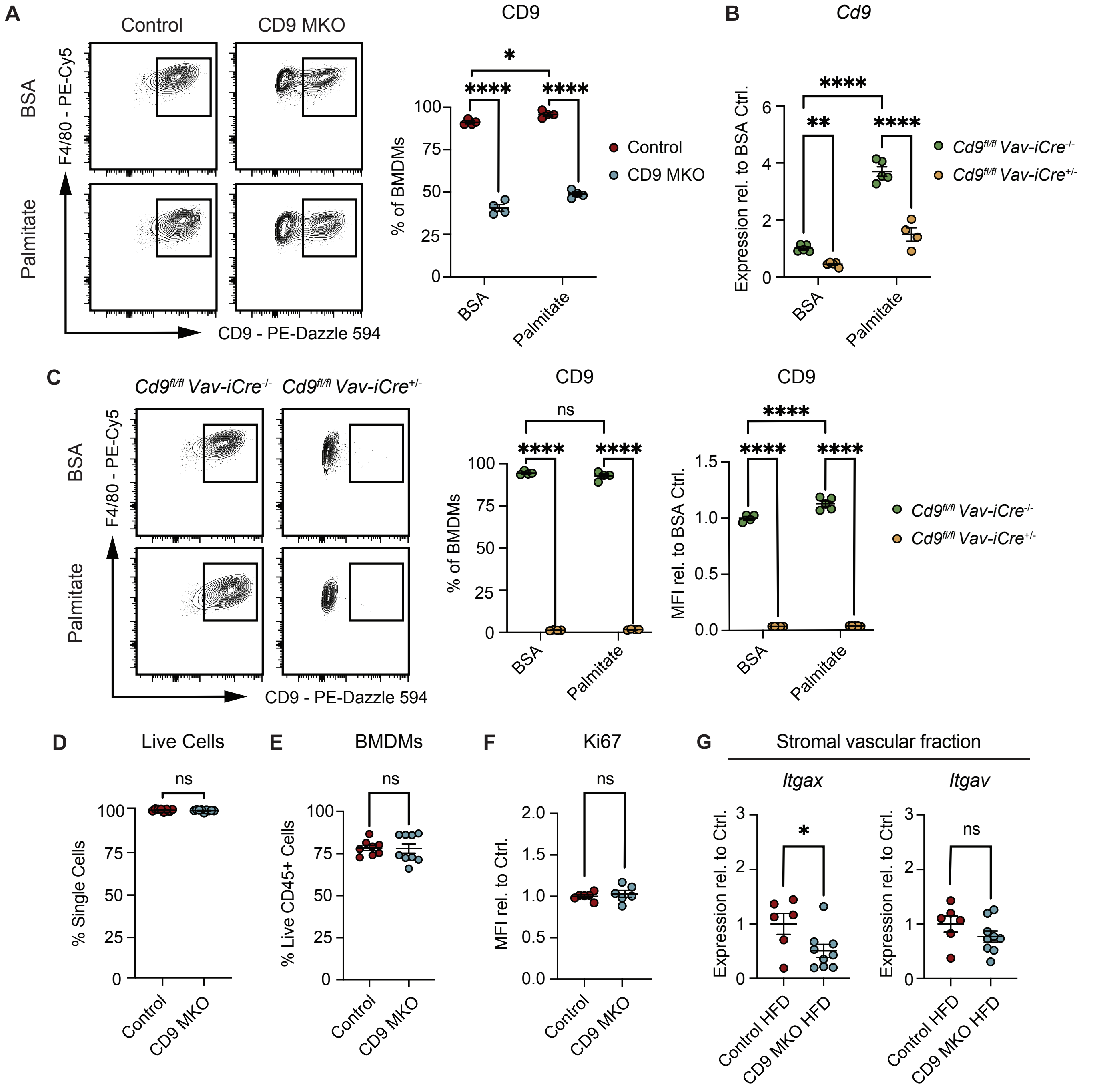

Supplementary Figure 6: Metabolic activation of macrophages regulates CD9 expression. (**A-F**) Bone marrow-derived macrophages (BMDMs) were polarized with BSA or palmitate for 24 hours. Flow cytometry of BMDMs was performed. BMDMs are gated as Live CD45$^+$ CD11b$^+$ F4/80$^+$ CD64$^+$ cells. (**A**) Representative flow plots and quanitifcation of surface expression of CD9 shown as a percentage of total BMDMs isolated from Control (*Cd9$^{fl/fl}$)* or CD9 MKO (*Cd9$^{fl/fl}$ LysMCre*$^{+/-}$) mice (n=4). (**B**) Expression of *Cd9* in BMDMs from *Cd9$^{fl/fl}$ Vav-iCre*$^{-/-}$ or *Cd9$^{fl/fl}$ Vav-iCre*$^{+/-}$ mice was assessed by qPCR (n=4-5). Shown as expression relative to BSA Controls (Ctrl.). (**C**) Representative flow plots and quantification of surface expression of CD9 in BMDMs from *Cd9$^{fl/fl}$ Vav-iCre*$^{-/-}$ or *Cd9$^{fl/fl}$ Vav-iCre*$^{+/-}$ mice shown as a percentage of total BMDMs or as relative MFI compared to BSA Controls (n=4-5). (**D-F**) BMDMs from Control or CD9 MKO mice polarized with BSA for 24 hours. (**D**) Frequency of live cells (n=8-9). (**E**) Frequency of CD64$^+$ F4/80$^+$ cells from BMDM cultures (n=8-9). (**F**) Mean fluorescence intensity (MFI) of Ki67 is shown as MFI relative to Control (Ctrl.) BMDMs (n=6). (**G**) Relative expression of *Itgax* and *Itgav* in epididymal white adipose tissue (eWAT) stromal vascular fractions isolated from Control or CD9 MKO mice fed a high fat diet (n=6-9). Representative data from 3 independent experiments (**A-C**) or pooled data from 2-3 independent experiments (**D-G**). Data presented as mean ± SEM. Statistics: Two-way ANOVA with Fisher's LSD test (**A-C**) or unpaired Student's t-test (**D-G**). ns = not significant. $^*p < 0.05$, $^{**}p < 0.01$, $^{****}p < 0.0001$.

Chini et al.- Supplemental. Figure 7

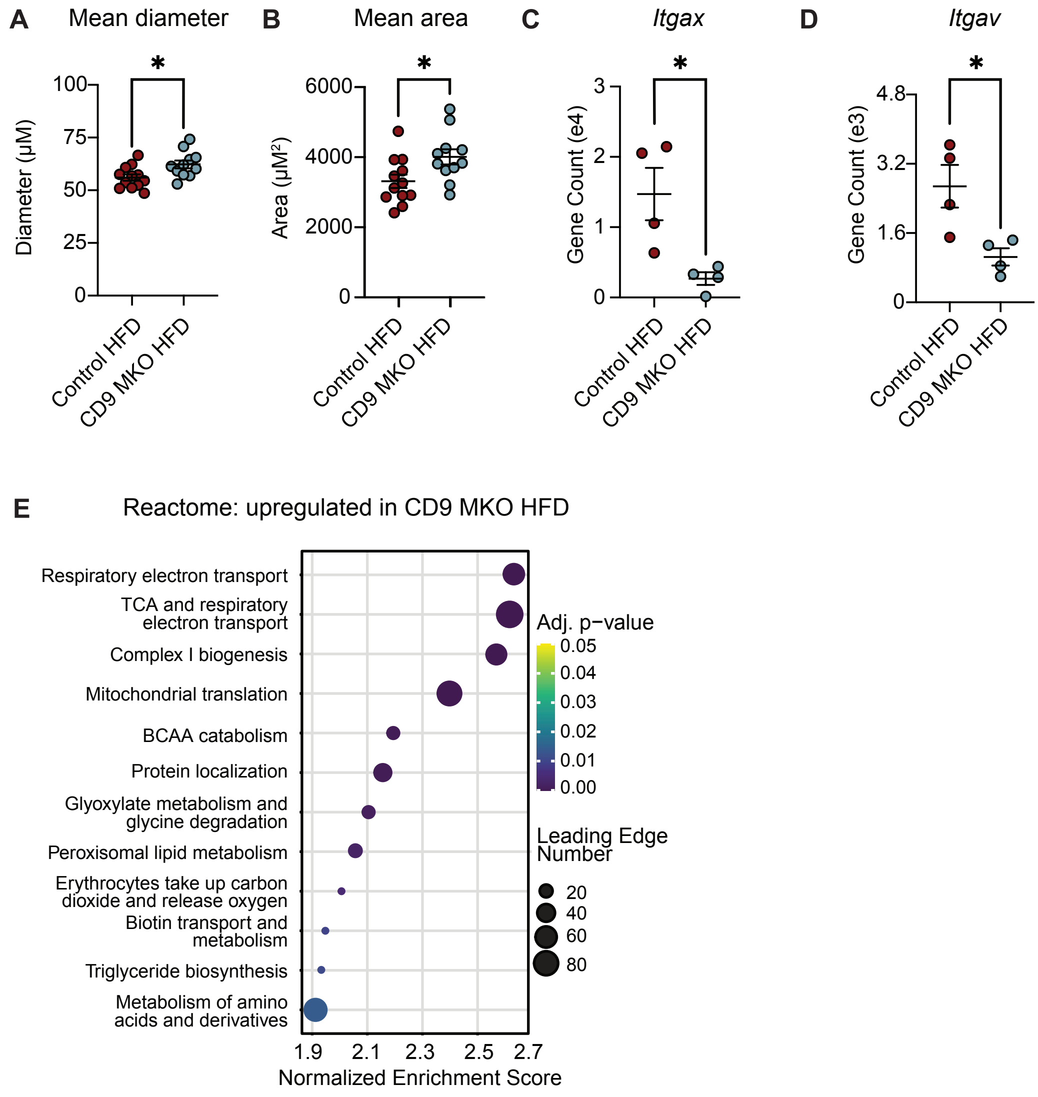

Supplementary Figure 7: Loss of myeloid-intrinsic CD9 leads to alterations in eWAT remodeling. Epididymal white adipose tissue (eWAT) was collected from male Control (*Cd9*$^{fl/fl}$) or CD9 MKO (*Cd9*$^{fl/fl}$ *LysMCre*$^{+/-}$) mice fed a high-fat diet (HFD) for 12 weeks. (**A, B**) Mean diameter (**A**) and area (**B**) of adipocytes from eWAT Hematoxylin & Eosin staining images shown in **Figure 4B** (n=11-12). (**C** and **D**) Gene count of *Itgax* (**C**) and *Itgav* (**D**) in eWAT from Control and CD9 MKO mice (n=4). (**E**) Pathway enrichment analysis showing most significant (by normalized enrichment score) nonredundant pathways upregulated in eWAT from CD9 MKO mice fed a HFD compared to controls (n=4). Pooled data from 3 independent experiments (**A** and **B**) or data from one experiment (**C-E**). Data presented as mean ± SEM. Statistics: unpaired Student's t-test (**A-D**). *p < 0.05. Abbreviations: BCAA: Branched-Chain Amino Acids; TCA: Tricarboxylic Acid Cycle.

Chini et al.- Supplemental. Figure 8

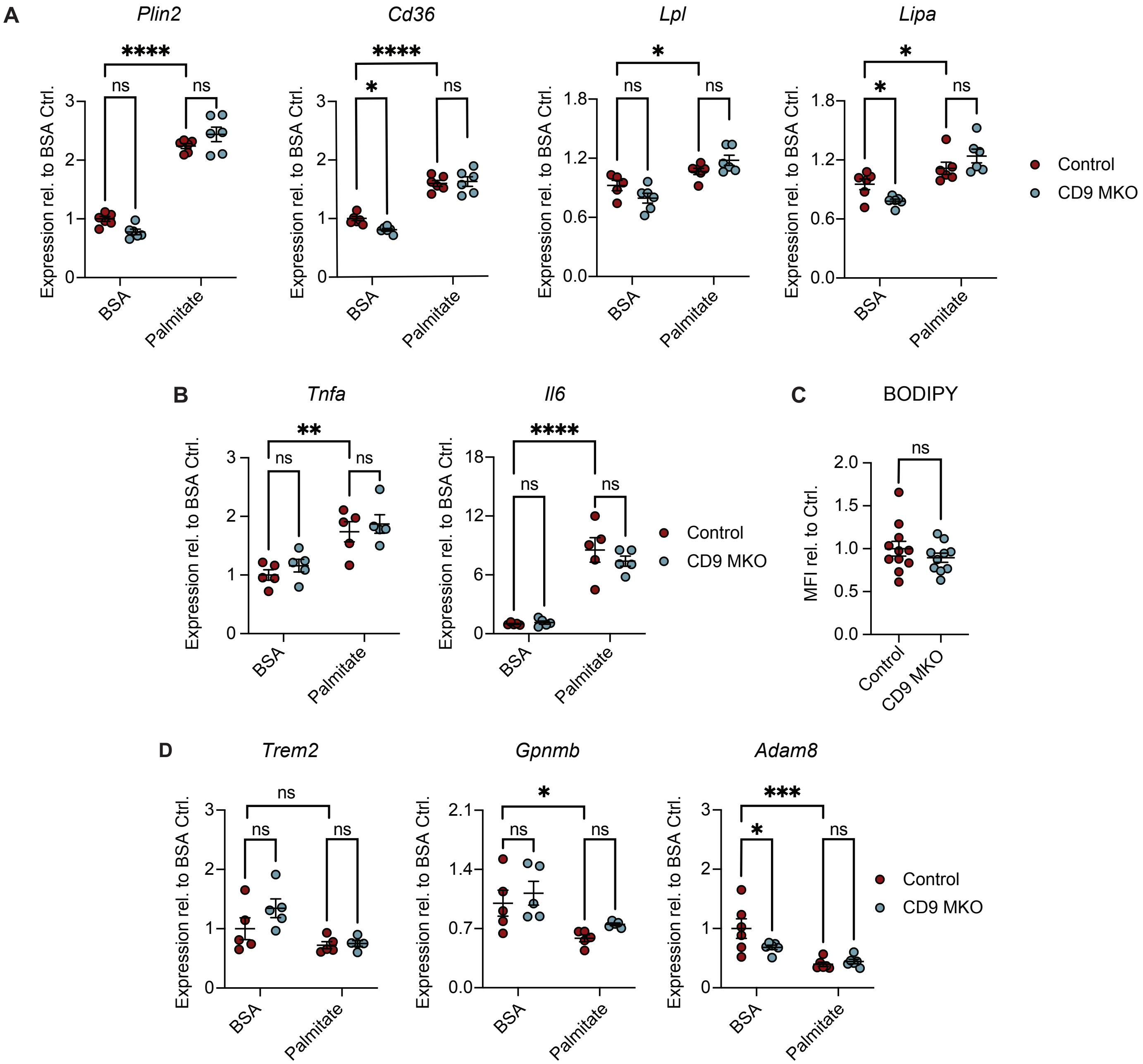

Supplementary Figure 8: CD9 does not regulate macrophage lipid metabolism in vitro. Bone marrow derived macrophages (BMDMs) isolated from Control (*Cd9*$^{fl/fl}$) or CD9 MKO (*Cd9*$^{fl/fl}$ *LysMCre*$^{+/-}$) mice were polarized with BSA or palmitate for 24 hours. (**A**) Expression of lipid metabolism genes (*Plin2*, *Cd36*, *Lpl*, and *Lipa;* n=5-6). (**B**) Expression of inflammatory cytokines (*Tnfa* and *Il6*; n=5). (**C**) BODIPY staining for intracellular lipids shown as Mean Fluorescence Intensity (MFI) relative to Controls (n=10-11). (**D**) Expression of *Trem2*, *Gpnmb*, and *Adam8* (n=5-6). Pooled (**C**) or representative data (**A, B, D**) from 3 independent experiments. qPCR of BMDMs is shown as ΔCt relative to *Hprt* normalized to BSA Controls. Data presented as mean ± SEM. Statistics: unpaired Student's t-test (**C**) or Two-way ANOVA with Fisher's LSD test (**A, B, D**). ns = not significant, *p < 0.05, **p < 0.01, ***p < 0.001, ****p < 0.0001.

Chini et al.- Supplemental. Figure 9

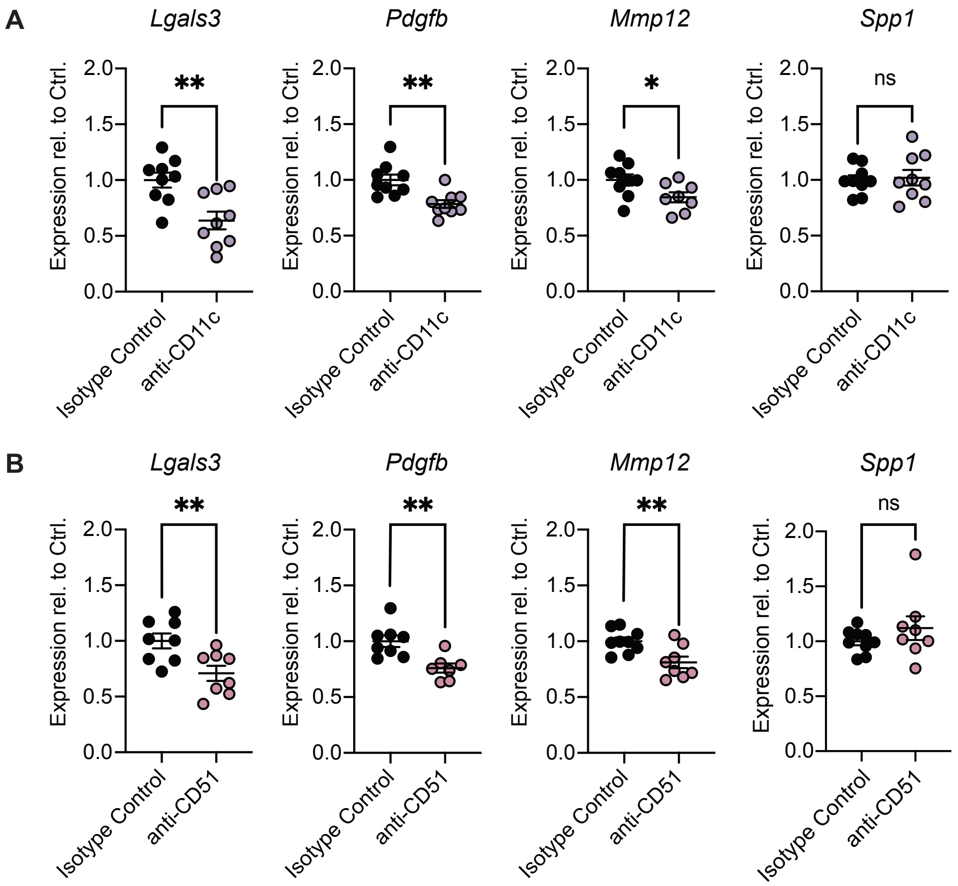

Supplementary Figure 9: CD11c and CD51 regulate macrophage expression of fibrosis-associated genes in vitro. Wild-type bone marrow-derived macrophages (BMDMs) were polarized with palmitate and treated with anti-CD11c (**A**) anti-CD51 (**B**), or isotype controls for 24 hours. Expression of fibrosis-associated genes *Lgals3*, *Pdgfb, Mmp12*, and *Spp1* was measured by qPCR. Data shown as ΔCt relative to *Hprt* normalized to isotype controls (n=7-9). Pooled data from 3 independent experiments. Data presented as mean ± SEM. Statistics: unpaired Student's t-test. ns = not significant, *p < 0.05, **p < 0.01.

Chini et al.- Supplemental. Figure 10

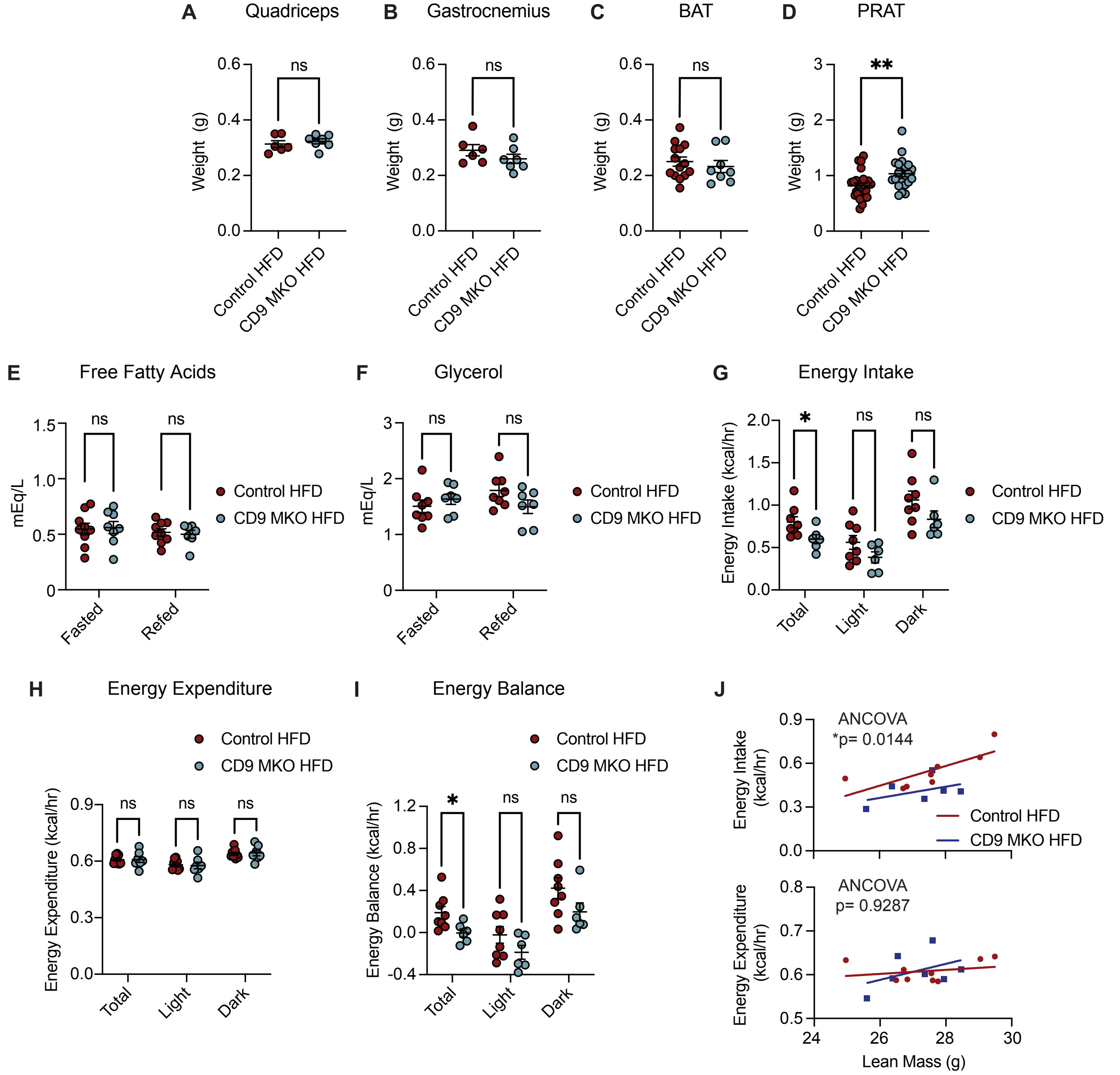

Supplementary Figure 10: Deletion of CD9 in myeloid cells alters visceral adiposity and energy intake during obesity. Control (*Cd9*$^{fl/fl}$) or CD9 MKO (*Cd9*$^{fl/fl}$ *LysMCre*$^{+/-}$) male mice fed a high fat diet (HFD) for 12 weeks. (**A-B**) Weight of quadriceps (**A**, n=6-7) or gastrocnemius (**B**, n=6-7) muscle. (**C**) Total weight of supraclavicular brown adipose tissue (BAT; n=8-14). (**D**) Weight of one side of perirenal white adipose tissue (PRAT) depot; n=22-26). (**E**) Plasma non-esterified free fatty acids levels following fasted (16 hours) or refed (4 hours) conditions (n=7-9). (**F**) Plasma glycerol levels following fasted (16 hours) or refed (4 hours) conditions (n=7-9). (**G-J**) Average energy intake (**G**), energy expenditure (**H**), and energy balance (**I**) in Control and CD9 MKO mice (n=6-9). (**J**) ANCOVA analysis for energy intake and energy expenditure (n=6-8). Pooled data from 2-6 independent experiments. Data presented as mean ± SEM. Statistics: unpaired Student's t-test (**A-D, G-I**), Two-way ANOVA with Fisher's LSD test (**E**, **F**) or ANCOVA analysis (**J**). ns = not significant, *p < 0.05, **p < 0.01.

Chini et al.- Supplemental. Figure 11

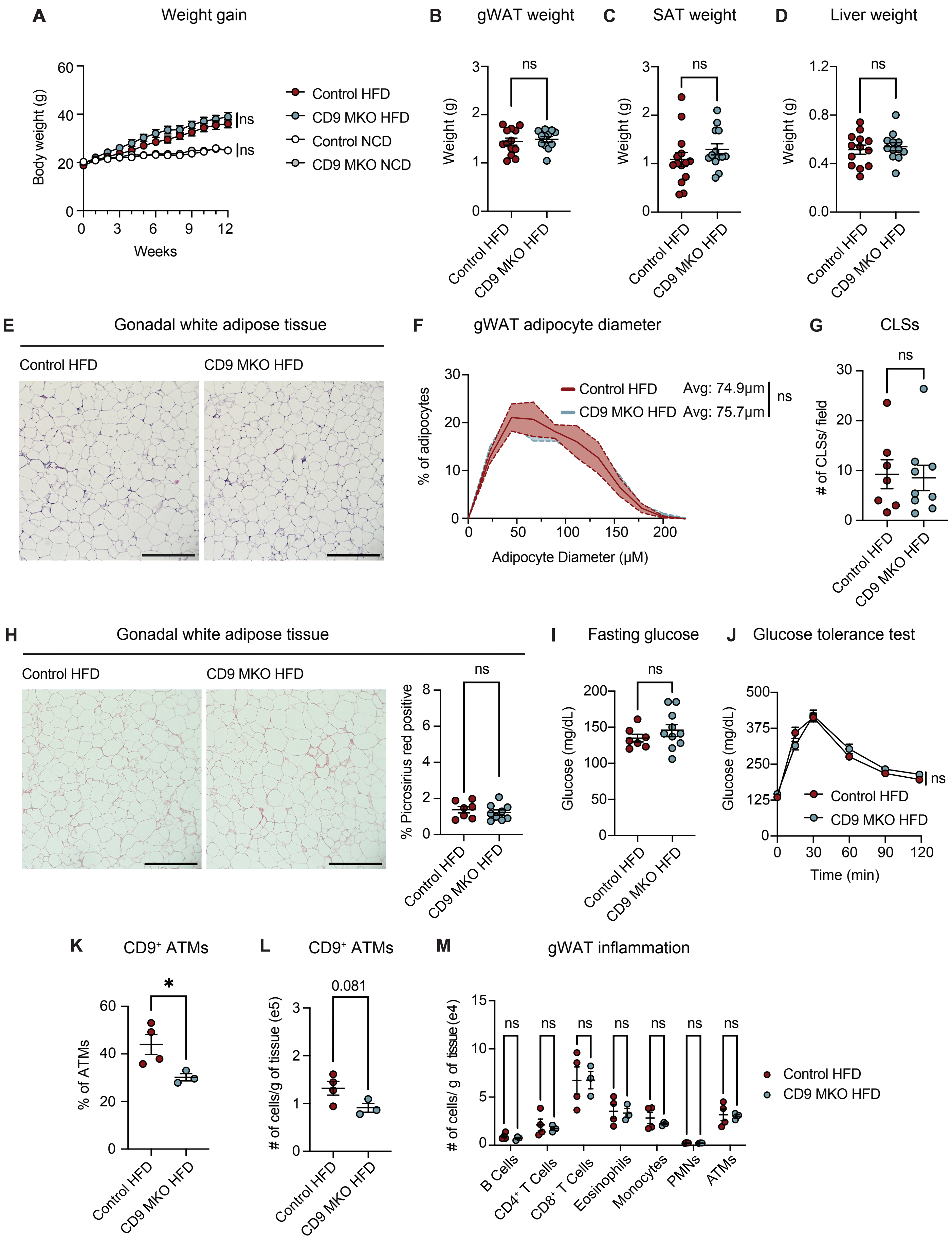

Supplementary Figure 11: Deletion of CD9 in myeloid cells leads to minimal metabolic changes in female mice. Female Control (*$Cd9^{fl/fl}$*) and CD9 MKO (*$Cd9^{fl/fl}$ LysMCre*$^{+/-}$) mice were placed on either a normal chow diet (NCD) or high-fat diet (HFD) for 12 weeks (**A**) Weekly body weights over 12 weeks (n=7-19). (**B-D**) Tissue weights in Control and CD9 MKO mice fed a HFD. (**B**) Visceral (perigonadal) white adipose tissue weight (gWAT; n=12-13). (**C**) Subcutaneous (inguinal) adipose tissue weight (n=13-14). (**D**) Liver (left lobe) weight (n=12-13). (**E**) Representative Hematoxylin & Eosin (H&E) staining of gWAT in Control or CD9 MKO HFD mice (n=7-9). Scale bar = 400µm. (**F**) Frequency distribution and mean adipocyte diameter calculated from H&E images in **E** (n= 7-9). (**G**) Total crown-like structures (CLSs) per field quantified from H&E images shown in **E** (n=7-9). (**H**) Representative images and quantification of picrosirius red staining of eWAT from Control and CD9 MKO mice (n=7-9). Scale bar = 400µm. (**I**) Glucose levels after a 16-hour fast in Control and CD9 MKO mice fed a HFD for 12 weeks (n=7-10). (**J**) Intraperitoneal glucose tolerance test (GTT) performed in Control and CD9 MKO female mice after 12 weeks of a HFD (n=7-9). (**K** and **L**) Frequency and number per gram of tissue of $CD9^+$ ATMs in gWAT (n=3-4). (**M**) Subsets of immune cells in gWAT of CD9 MKO and Control mice (n=3-4). Pooled data from 2-4 independent experiments (**A-J**) or representative data from 3 independent experiments (**K-M**). Data presented as mean ± SEM. Statistics: Two-Way ANOVA with multiple comparisons (**A, J**) or unpaired Student's t-test (**B-D, F-I, K-M**). ns = not significant, *$p < 0.05$.

Table S1: Flow cytometry antibodies and other reagents					
**Target**	**Fluorophore**	**Concentration**	**Clone**	**Supplier**	**Catalog #**
B220	BV750	1:500	RA3-6B2	Biolegend	103261
BODIPY 493/503	n/a	1:2400	n/a	Invitrogen	D3922
CD11b	BV785	1:500	M1/70	BioLegend	101243
CD11c	PE-Cy5.5	1:500	N418	BioLegend	117327
CD16/CD32 (Fc Block)	n/a	1:50	2.4G2	BD	553142
CD19	PE-Cy5	1:500	6D5	BioLegend	115510
CD4	APC	1:500	GK1.5	BioLegend	100411
CD4	PE-Cy5	1:500	GK1.5	BioLegend	100410
CD45	BUV395	1:500	30-F11	BD	564279
CD45	BV510	1:100	A20	BioLegend	110741
CD51	BV421	1:500	RMV-7	BD	740062
CD64	BV711	1:500	X54-5/7.1	BioLegend	139311
CD64	PE-Dazzle	1:500	X54-5/7.1	BioLegend	139304
CD8a	Pacific Blue	1:500	53-6.7	BD	558106
CD8a	PE-Cy5	1:500	53-6.7	BioLegend	100710
CD9	BV421	1:500	RMV-7	Fisher Scientific	752985
CD9	PE	1:500	MZ3	BioLegend	124806
CD9	PE-Cy7	1:500	MZ3	BioLegend	124815
CD9	PE-Dazzle 594	1:500	MZ3	BioLegend	124821
F4/80	APC	1:500	T45-2342	BD	566787
F4/80	BV605	1:500	BM8	BioLegend	123133
F4/80	PC-Cy5	1:500	BM8	BioLegend	123111
F4/80	PerCP-Cy5.5	1:250	BM8	BioLegend	123128
LIVE/DEAD™ blue	n/a	1:2400	n/a	Invitrogen	L23105
Ly6C	APC	1:500	HK1.4	BioLegend	128016
Ly6C	BV510	1:500	HK1.4	BioLegend	128033
Ly6G	AF700	1:500	1A8	BioLegend	127622
Ly6G	BV650	1:500	1A8	BioLegend	127641
NK-1.1	PE-Cy5	1:500	PK136	BioLegend	108715
Siglec-F	APC-Cy7	1:500	E50-2440	BD	565527
TER-119	PE-Cy5	1:500	TER-119	BioLegend	116209
Ki67	APC	1:500	16A8	BioLegend	652405

Table S2: qPCR primer sequences		
**Gene**	**Forward**	**Reverse**
Actb	GCAGCGATATCGTCATCCATG	AGCTTCTTTGCAGCTCCTTC
Adam8	TCCCAAGAGCATAGCTCAGA	TTGCCCCATGTGAAACAGTA
Adgre1	ACCACAATACCTACATGCACC	AAGCAGGCGAGGAAAAGATAG
B2m	TCTGGTGCTTGTCTCACTGAC	GCAGTTCAGTATGTTCGGCTTC
Cd36	GTTGACCTGCAGTCGTTTTG	TGAAGGCTTACATCCAAATGAA
Cd68	GTGTAGTTCCCAAGAGCCCC	CCACAGTTTCTCCCACCACA
Cd9	GGCGAATATCACCAAGAGGA	AGAGTCCCAGTGCATGCTG
Gpnmb	GCTTTGTCTACGTCTTTCACACA	CTGAACACCGACCCAGTTTT
Hprt	TCAGTCAACGGGGGACATAA	GGGGCTGTACTGCTTAACCAG
Il6	CCATAGCTACCTGGAGTACATG	TGGAAATTGGGGTAGGAAGGAC
Itgav	CCAGCCCATTGAGTTTGATT	TCCAGTGGGTCATCTTTGG
Itgax	CAGAACTTCCCAACTGCACA	TCAGGAACACGATGTCTTGG
Lgals3	CTGCTGGCCCTTATGGTGT	ATGACTCCTCCAGGCAAGG
Lipa	GCAAGTGGTCCGATTCCCTT	GGAGCAAAGCAGGCTCAGTA
Lpl	CCCTAAGGACCCCTGAAGAC	GGCCCGATACAACCAGTCTA
Mmp12	GGATGAAGCGGTACCTCACT	ACATCCTCACGCTTCATGTC
Pdgfb	GCTCGGGTCATGTTCAAGTC	CCTGCTGCACAGAGACTCC
Plin2	TCCCTCAGCTCTCCTGTTAG	TGACATAAGCGGAGGACACA
Rpl13a	TCCGATAGTGCATCTTGGCC	CAAGGTTGTTCGGCTGAAGC
Sdha	CTCAACCACAGAGGCAGGAG	CGAGCTGCATTTGGCCTTTC
Spp1	TGCTGTGTCCTCTGAAGAAAA	TGGCTTTCATTGGAATTGCT
Tnfa	GGTGCCTATGTCTCAGCCTC	GCTCCTCCACTTGGTGGTTT
Trem2	CTCCACCAGTTTCTCCTGCT	AGTGCTTCAAGGCGTCATAAGT
