# Supplementary figures and images for: CD9 regulates macrophage-mediated remodeling of adipose tissue in obesity
Source: JCI Insight. 2026 Feb 10;11(6):e193837. doi: 10.1172/jci.insight.193837 (PMC13043082; doi:10.1172/jci.insight.193837)

Chini et al. Full Unedited Blot (Supplemental Figure 1B)

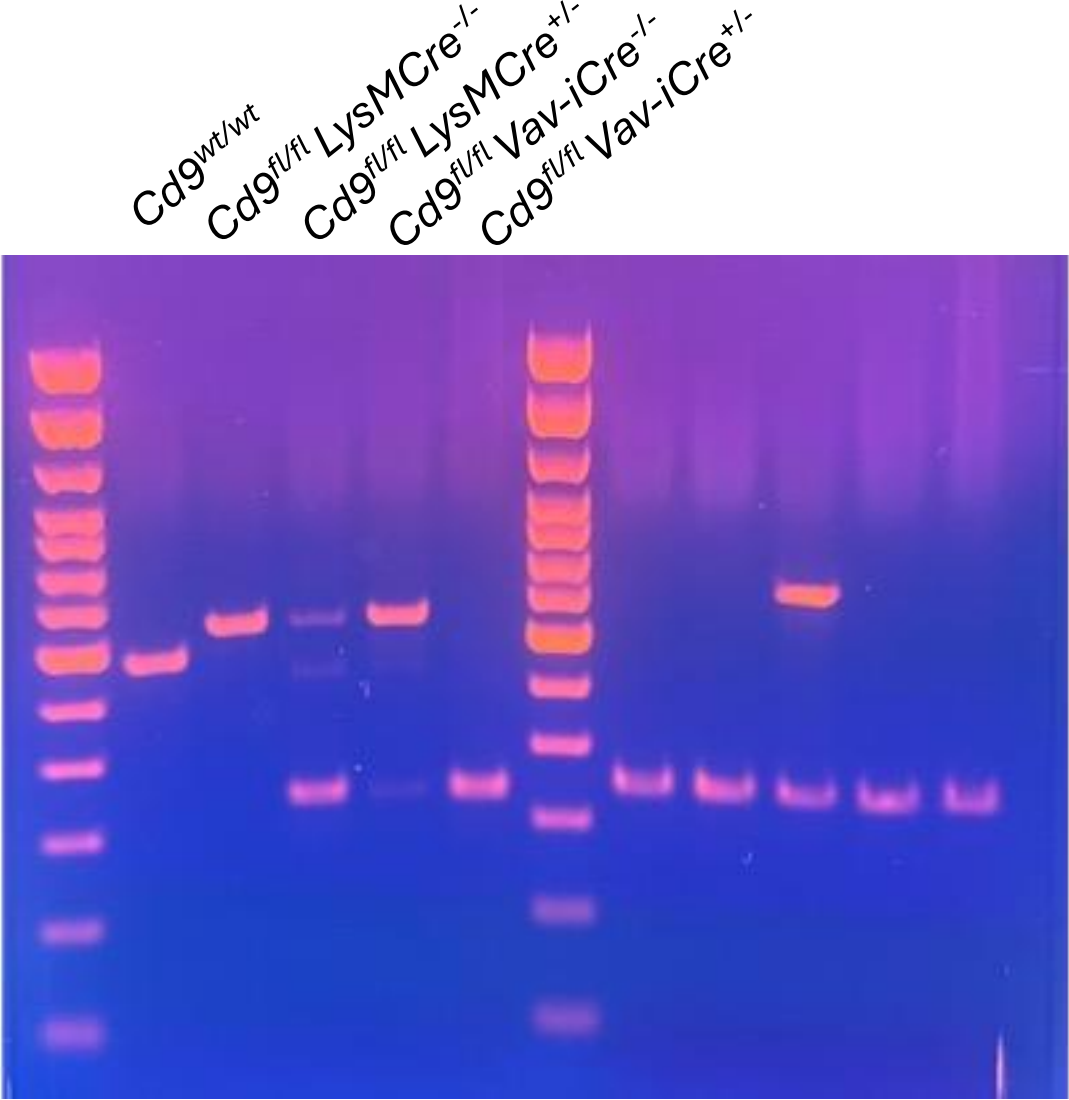

Supplement: Unedited blot and gel images [file jciinsight-11-193837-s198.pdf]
